# Comparison of conventional scoring systems to machine learning models for the prediction of major adverse cardiovascular events in patients undergoing coronary computed tomography angiography

**DOI:** 10.3389/fcvm.2022.994483

**Published:** 2022-10-26

**Authors:** Seyyed Mojtaba Ghorashi, Amir Fazeli, Behnam Hedayat, Hamid Mokhtari, Arash Jalali, Pooria Ahmadi, Hamid Chalian, Nicola Luigi Bragazzi, Shapour Shirani, Negar Omidi

**Affiliations:** ^1^Tehran Heart Center, Tehran University of Medical Science, Tehran, Iran; ^2^Biomedical Engineering and Physics Department, School of Medicine, Shahid Beheshti University of Medical Sciences, Tehran, Iran; ^3^Division of Cardiothoracic Imaging, Department of Radiology, University of Washington, Seattle, WA, United States; ^4^Laboratory for Industrial and Applied Mathematics (LIAM), Department of Mathematics and Statistics, York University, Toronto, ON, Canada; ^5^Department of Cardiovascular Imaging, Tehran Heart Center, Tehran University of Medical Sciences, Tehran, Iran

**Keywords:** coronary computed tomography angiography, machine learning, coronary artery calcium scores, major adverse cardiovascular events, conventional scoring

## Abstract

**Background:**

The study aims to compare the prognostic performance of conventional scoring systems to a machine learning (ML) model on coronary computed tomography angiography (CCTA) to discriminate between the patients with and without major adverse cardiovascular events (MACEs) and to find the most important contributing factor of MACE.

**Materials and methods:**

From November to December 2019, 500 of 1586 CCTA scans were included and analyzed, then six conventional scores were calculated for each participant, and seven ML models were designed. Our study endpoints were all-cause mortality, non-fatal myocardial infarction, late coronary revascularization, and hospitalization for unstable angina or heart failure. Score performance was assessed by area under the curve (AUC) analysis.

**Results:**

Of 500 patients (mean age: 60 ± 10; 53.8% male subjects) referred for CCTA, 416 patients have met inclusion criteria, 46 patients with early (<90 days) cardiac evaluation (due to the inability to clarify the reason for the assessment, deterioration of the symptoms vs. the CCTA result), and 38 patients because of missed follow-up were not enrolled in the final analysis. Forty-six patients (11.0%) developed MACE within 20.5 ± 7.9 months of follow-up. Compared to conventional scores, ML models showed better performance, except only one model which is eXtreme Gradient Boosting had lower performance than conventional scoring systems (AUC:0.824, 95% confidence interval (CI): 0.701–0.947). Between ML models, random forest, ensemble with generalized linear, and ensemble with naive Bayes were shown to have higher prognostic performance (AUC: 0.92, 95% CI: 0.85–0.99, AUC: 0.90, 95% CI: 0.81–0.98, and AUC: 0.89, 95% CI: 0.82–0.97), respectively. Coronary artery calcium score (CACS) had the highest correlation with MACE.

**Conclusion:**

Compared to the conventional scoring system, ML models using CCTA scans show improved prognostic prediction for MACE. Anatomical features were more important than clinical characteristics.

## Graphical Abstract

Comparison of conventional scoring systems to machine learning models for prediction of major adverse cardiovascular events in patients undergoing coronary computed tomography angiography. 
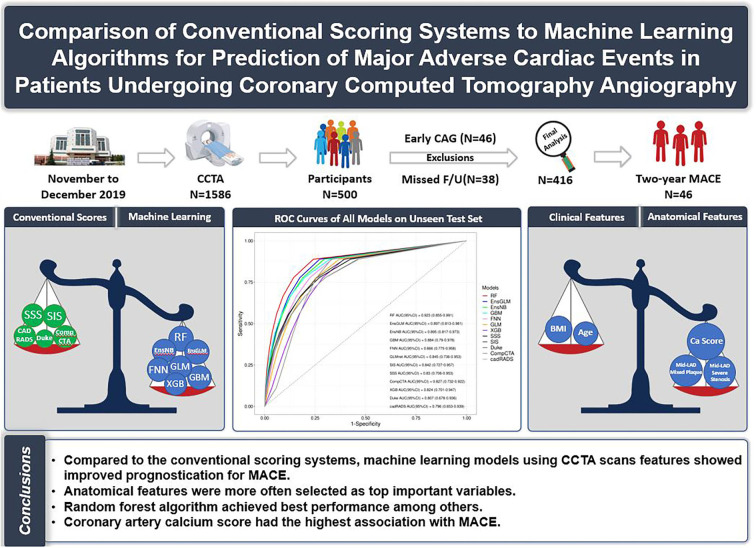

## Introduction

The progressive improvement in coronary computed tomography angiography (CCTA) techniques is an ongoing evolution to better identify coronary artery disease (CAD) and its outcomes. Different conventional scoring systems have been developed for the risk stratification of adverse events. One of them, coronary artery calcium scoring (CACS) using computed tomography is clinically valuable for estimating CAD (1, 2). Some other conventional scoring methods, such as CAD vessel score based on the number of vessels and CAD severity based on the severity of obstruction, are still being used (3). Other scoring systems based on anatomical location include segment involvement score (SIS) and segment stenosis score (SSS) (3, 4). After that, Duke criteria gave more weight to the proximal portion of the left anterior descending artery (LAD) than the other scoring systems (5). The coronary artery disease reporting and data system (CAD-RADS) is another scoring system designed to create a standard method for the findings of CCTA (6). Finally, the comprehensive CTA score (Comp.CTAS) was organized to show that the integration of plaque extent, location, and composition in a comprehensive model may improve risk stratification (7). Despite the gradual improvement in the conventional scoring system, the main limitation of all of them was that they were only developed based on anatomical features. Different factors encompassing CACS, anatomical, and clinical factors have prognostic value in the prediction of MACE (4, 8). ML methods such as boosted ensemble algorithm consisting of only anatomical or clinical features have shown promising results in distinguishing patients with and without MACE compared to some conventional scoring systems (9, 10). While great work has been concentrated on developing scores based on the so-called conventional scores, ML methods have not been investigated as much as the conventional scores, and the efficacy of a mixture of all factors has been missing in the literature (10, 11). Current literature on the ML-based prediction of MACE has only taken into account some of the prognostic variables at the same time, whereas this article has considered all of the variables simultaneously by employing the ML methods (10). Another superiority of this article is that several machine learning models have been created and compared to the conventional scoring systems, while other ML-based studies only consist of one ML model (9, 11).

The aim of the study is to develop ML methods considering multiple variables with possible prognostic values to improve the prediction of MACE based on CCTA data.

## Materials and methods

### Study description

We conducted a cross-sectional study on patients between 40 and 90 years old from November 2019 to December 2019 and referred to our institution for coronary computed tomography angiography (CCTA) evaluation. This period was limited because of introducing COVID-19 as a specific global human disaster. During this period, 1,586 patients underwent CCTA. Exclusion criteria were set as the following: poor image quality (including motion artifact, low signal-to-noise ratio), history of coronary artery bypass graft surgery (CABG), history of percutaneous coronary intervention (PCI), and history of recent (30 days) ACS (including myocardial infarction and unstable angina).

In the emergency department of our center, patients with low risk of unstable angina according to thrombolysis in myocardial infarction (TIMI) score (0–1 score) and a couple of negative troponins were scheduled for noninvasive strategies in the next 24–72 h. If these patients came back for further investigation in 1 day up to 1 month, they were excluded from our study. Moreover, some of these patients referred for CCTA returned for further evaluation after a month, and they underwent CCTA as stable ischemic heart disease; therefore, we enrolled this group of patients in our study.

Some studies indicated that a high coronary calcium score might overestimate the stenosis (12). Based on our experience 128-Slice Dual-Source CT scanner patients with CACS of more than 1,200 should be excluded due to lowering the accuracy of the study.

After applying exclusion criteria (*N* = 322), a total of 500 of 1,264 patients were recruited consecutively based on their coronary artery calcium score (CACS), and divided into five groups (CACS: 0, 1–10, 10–100, 100–400, >400). Each group was supposed to have an equal number of patients. However, due to the COVID-19 restriction, the data gathering process was stopped after 2 months. Until then, we reached the goal of at least 100 patients in four primary groups. And only in CACS >400 groups, 87 patients were collected. Since the number of patients in our study was limited by COVID-19 and the probability of missed follow-up, in order to prevent a further drop in the number of patients, to reach 500 cases, we decided to recruit 13 more patients which were distributed in the groups with CACS lower than 400. The distribution of cases in the four defined groups is shown in Table 1.

**Table 1:** The baseline demographic and clinical characteristics of the patients who underwent coronary computed tomography angiography.

**Variable**	**Calcium coronary artery score category (*****N*** = **500)**					***P***
	**0** **(*N* = 102)**	**1–10** **(*N* = 103)**	**11–100** **(*N* = 103)**	**101–400** **(*N* = 105)**	**>400** **(*N* = 87)**	
Age (years)	53.03 ± 7.97	59.01 ± 10.63	61.70 ± 8.19	63.60 ± 9.54	63.75 ± 9.00	<0.001
Body mass index (kg/m^2^)	28.29 ± 4.19	28.23 ± 4.18	27.83 ± 4.20	28.18 ± 4.03	28.51 ± 4.85	0.869
Ejection fraction (%)	55.22 ± 4.96	55.06 ± 5.34	54.14 ± 6.74	54.17 ± 6.47	53.66 ± 4.73	0.436
Gender [*N* (%)]						0.663
Male	51 (50.0)	56 (54.4)	52 (50.5)	58 (55.2)	52 (59.8)	
Female	51 (50.0)	47 (45.6)	51 (49.5)	47 (44.8)	35 (40.2)	
Diabetes mellitus [*N* (%)]	22 (21.6)	31 (30.1)	27 (26.2)	35 (33.3)	29 (33.3)	0.293
Hypertension [*N* (%)]	46 (45.1)	51 (49.5)	64 (62.1)	67 (63.8)	57 (65.5)	0.008
Dyslipidemia [*N* (%)]	55 (53.9)	61 (59.2)	68 (66.0)	63 (60.0)	60 (69.0)	0.220
Renal failure [*N* (%)]	1 (1.0)	6 (5.8)	5 (5.8%)	6 (5.7)	5 (5.7)	0.306
Family history of CAD* [*N* (%)]	39 (38.2)	47 (45.6)	38 (36.9)	37 (35.2)	31 (35.6)	0.541
Cigarette smoker [*N* (%)]	12 (11.8)	15 (14.6)	26 (4.9)	14 (13.3)	18 (20.7)	0.056
Anti-platelet [*N* (%)]	50 (49.0)	57 (55.3)	56 (54.4)	68 (64.8)	59 (67.8)	0.047
Statin [*N* (%)]	46 (45.1)	53 (51.5)	60 (58.3)	58 (55.2)	61 (70.1)	0.011
B-blocker [*N* (%)]	44 (43.1)	45 (43.7)	53 (51.5)	47 (44.8)	46 (52.9)	0.508
ACEI/ARB** [*N* (%)]	33 (32.4)	44 (42.7)	49 (47.6)	58 (55.2)	53 (60.9)	0.001
Left main (LM) coronary artery [*N* (%)]	0 (0.0)	0 (0.0)	0 (0.0)	4 (3.8)	2 (2.3)	0.013
CAD severity [*N* (%)]						<0.001
Normal	34 (33.3)	0 (0.0)	0 (0.0)	0 (0.0)	0 (0.0)	
Non-obstructive	56 (54.9)	83 (80.6)	71 (68.9)	41 (39.0)	12 (13.8)	
Obstructive	9 (8.9)	15 (14.6)	20 (19.4)	32 (30.5)	26 (29.9)	
Severe obstructive	3 (2.9)	5 (4.8)	12 (11.7)	32 (30.5)	49 (56.3)	
CAD vessel score [*N* (%)]						<0.001
Normal	34 (33.3)	0 (0.0)	0 (0.0)	0 (0.0)	0 (0.0)	
Mild CAD	56 (54.9)	83 (80.6)	71 (68.9)	41 (39.1)	12 (13.8)	
Single-vessel CAD	11 (10.8)	17 (16.5)	26 (25.2)	36 (34.3)	32 (36.8)	
Two-vessel CAD	1 (1.0)	2 (1.9)	5 (4.9)	16 (15.2)	28 (32.2)	
Three-vessel CAD /LM	0 (0.0)	1 (1.0)	1 (1.0)	12 (11.4)	15 (17.2)	

**ACEI/ARB, angiotensin-converting enzyme inhibitors/angiotensin II receptor blockers; *CAD, coronary artery disease.

**Figure 1:**
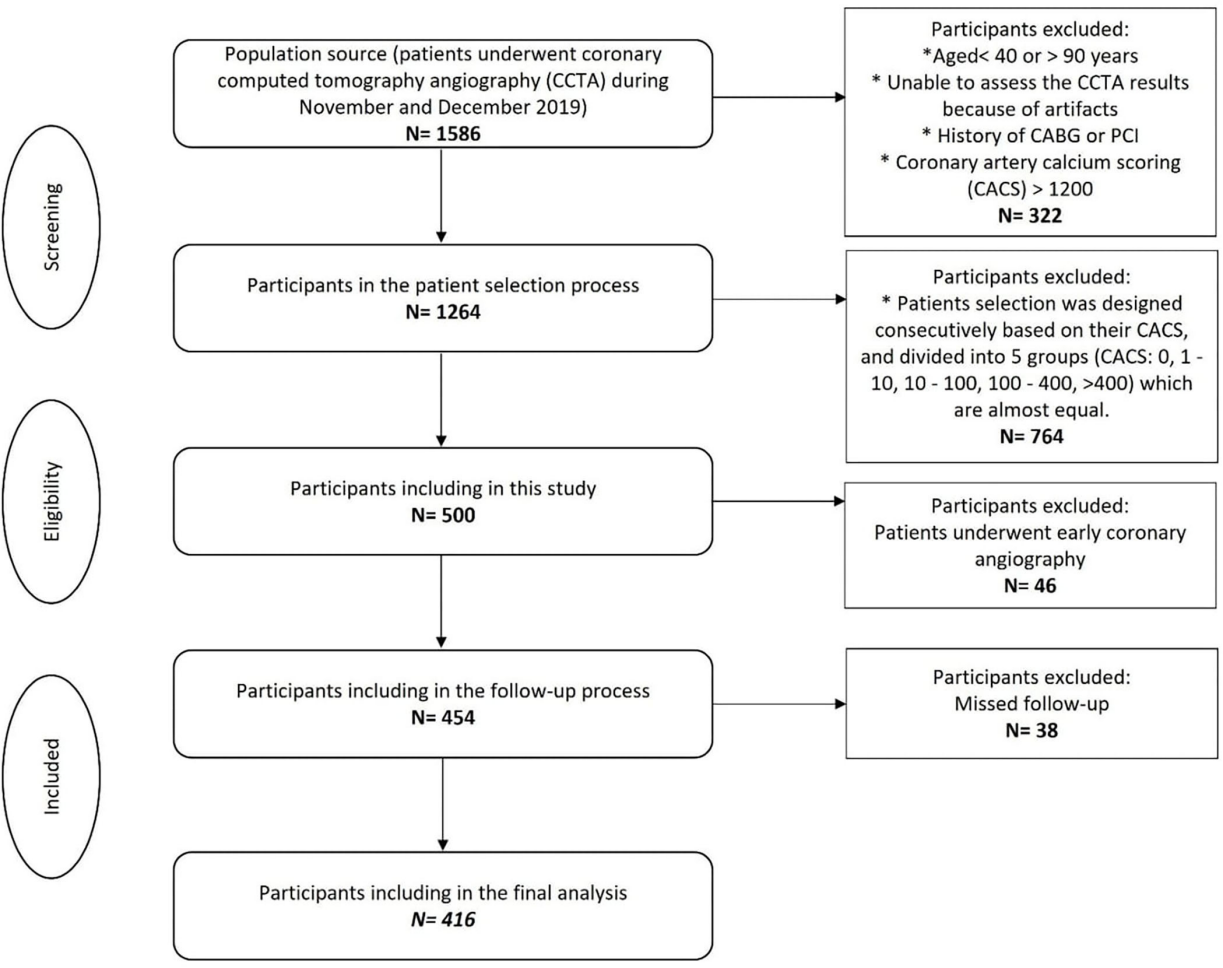
Flowchart of the participant's selection. CACS, Coronary artery calcium scoring; CCTA, Coronary computed tomography angiography. *Indicated as bullet point.

Patients who underwent invasive coronary angiography (ICA) within less than 90 days after CCTA did not enter the final analysis due to the inability to clarify whether the reason for ICA was an exacerbation of the symptoms or just because of the CCTA result. Early coronary evaluation (only ICA/PCI/CABG <90 days) was considered exclusion criteria. Among 500 participants, 46 patients were excluded due to early coronary angiography, and 38 participants were also excluded because of missed follow-up. Finally, 416 participants enrolled for the final analysis (Figure 1).

All participants provided written informed consent prior to the inclusion and the investigation conformed to the principles outlined in the Declaration of Helsinki. Approval for the study protocol was obtained from the institutional ethics committee of Tehran Heart Center (Ethical Code: IR.TUMS.THC.REC.1399.022).

### Data gathering

The patients' clinical characteristics and demographic information such as age, gender, body mass index (BMI), ejection fraction (EF), diabetes mellitus (DM), hypertension, dyslipidemia, renal failure, family history of CAD, and cigarette smoking were collected from the CCTA data bank. Cigarette smokers were classified as never or ever-users. Also, a history of the anti-platelet, statins, beta-blockers, and angiotensin-converting enzyme inhibitors (ACEIs)/ angiotensin II receptor blockers (ARBs), was gathered.

### Definitions of variables

Participants were considered to have DM if the fasting blood glucose was ≥126 mg/dL or 2-h postprandial blood glucose was ≥200 mg/dL or taking anti-diabetic medications (13). Hypertension was defined as mean systolic blood pressure ≥140 mmHg or mean diastolic blood pressure ≥90 mmHg or the use of antihypertensive medication (14). Renal failure was defined based on the presence of kidney damage or glomerular filtration rate <60 ml/min/1.73 m^2^ for ≥3 months at any time (15). Body mass index (BMI) [calculated as weight (kilogram (kg)] divided by the square root of height (meter [m]) was grouped as underweight (BMI <18.5 kg/m^2^), (18.5 ≤ BMI <25 kg/m^2^), overweight (25 ≤ BMI <30 kg/m^2^), and obese (BMI ≥30 kg/m^2^) according to the World Health Organization recommendations (16).

### CCTA procedure and findings

Our center is a specialized tertiary referral center for the management of cardiovascular disease; with more than 9000 CCTA performed annually. Siemens SOMATOM Definition Flash 128-Slice Dual-Source CT scanner was used to perform electrocardiogram-gated CCTA. The acquisition protocol is consistent with the Society of Cardiovascular Computed Tomography guidelines (17). This protocol is individualized for each patient either automatically or manually. Intravascular access was established using the facility's protocol and adequate flow should be ascertained before injection. Eighteen-gauge catheters are often necessary for adults. Iodine concentration is 270–400 (mgIodine/cc) and an injection rate between 5 and 7 cc/s should be used by the injector. The slice thickness was set to 0.6 and reconstruction kernel was set to 26 (17). A target heart rate for coronary CTA was set at 60 bpm and beta-blocker was considered a first line for achieving the heart rate. CACS was analyzed by the Agatston score (18). The CCTA results were reviewed by an expert cardiologist and a radiologist independently. In a disagreement situation, an in-person meeting was formulated between them for consensus. If that meeting was not conclusive, the third person blindly evaluate CCTA and made a final decision independently. Several conventional scores were used for the assessment of coronary artery stenosis, described as follows: CACS, SSS, SIS, Duke index, CAD-RADS, and Comp.CTAS.

### Study endpoint and follow-up

In this study, the endpoint was considered MACE including all-cause mortality, non-fatal myocardial infarction (MI), late coronary revascularization (PCI or CABG ≥ 90 days), and hospitalization for unstable angina or heart failure. A dedicated physician or staff performed patient follow-up for about 2 years. All the participants were followed for detecting the primary endpoint. This process was done by either phone interviews or reviewing the medical records for about 2 years.

### Conventional statistical analysis

Continuous data were described using mean with standard deviation (SD) or median with 25th and 75th percentiles for normally and skew-distributed variables, respectively. The normality of the variables was checked using histogram charts and descriptive measures, as well as the Kolmogorov test. Number and frequencies (%) were used to express categorical variables. IBM SPSS Statistics for Windows, version 25 (IBM Corp., Armonk, N.Y., USA, https://www.ibm.com) was used to conduct statistical analyses. The overall performance of the prediction model on the cross-validation dataset and unseen test set was assessed by calculating the area under the curve (AUC) from the receiver operating characteristic (ROC) curve analysis along with 95% confidence intervals (CIs). A *p* < 0.05 was considered statistically significant.

### Machine learning

#### Analysis foundation

The programming language used for machine learning and survival analysis was R software (version: 4.0.4). Integrated development environment used for analysis was RStudio (1.4.1106); integrated development environment for R. RStudio, PBC, Boston, MA URL http://www.rstudio.com/.

#### Machine learning algorithms

We encompassed linear and non-linear algorithms, including three trees-based [random forest (RF), gradient boosting machine (GBM), and eXtreme Gradient Boosting (XGB)], one neural network-based [feed-forward neural network (FNN)], two linear methods [generalized linear model (GLM) and support vector machine (SVM)], and another non-linear method [k-nearest neighbors (KNNs)]. After performing a spot check assessment of various algorithms, five algorithms with AUC> 0.7, namely, RF, GBM, XGB, FNN, and GLM, were selected to be utilized for artificial intelligence. To strengthen the final result, we performed two stacked ensemble algorithms in addition to the five mentioned above. One was powered by linear metalearner (GLM) and another one by non-linear metalearner (naive Bayes), importing all previously mentioned algorithms as input base learners.

#### Classification and regression tree algorithm

Based on CART, decision trees are the base learner for tree-based algorithms. Each decision tree consists of a root node which is the first node that contains all the observations in the training dataset. It starts by applying the predefined splitting rule, which uses the default number of random features in each split. This splitting rule tries to find the features that can divide observations into two best homogeneous groups based on their outcomes. This process continues in every daughter node with a random set of variables in each splitting point to reach the depth of terminal nodes or the splitting process cannot improve the discrimination of observations further. For classification problems, splitting rules are Gini impurity and information gain (entropy). Gini impurity calculates the probability of an observation being labeled incorrectly according to the proportion of the class in the population (here in each node), as described in Equation (1).


(1) $$\begin{array}{l} Gini\;impurity=1-\overset{n}{\underset{i}{\sum }}{\left(p_{i}\right)}^{2}\; \end{array}$$

where *p*_*i*_ is the proportion of a class in the total number of observations in the node.

Information gain tries to find how much information content is acquired by daughter nodes compared to their parent node. It calculates entropy of each node and subtracts their values from their parent node, and tries to maximize information gain (Equation 2).


(2) $$\begin{array}{l} Entropy=-\overset{n}{\underset{i}{\sum }}p_{i}\log_{2}p_{i}\; \end{array}$$

where *p*_*i*_ is the proportion of a class in the total number of observations in the node.

#### Bagging method

The decision tree has a low bias rate but a high variance rate. By combining the results of many decision trees, known as ensembling methods, one would avoid a high variance rate. By resampling the dataset with replacement (permutation), called bootstrapping, one tree is trained on each bootstrapped dataset, and hundreds to thousands of trees are created in a parallel fashion; then, the results of all trees are aggregated. This process is called the “bagging” method, derived from bootstrapping and aggregating.

#### Boosting method

Another way of utilizing the result of many trees is by learning from each tree error in a consecutive manner. Among boosting methods, gradient boosting methods use the loss function gradient of each tree to boost the next tree performance. One tree at each step is trained on the training dataset and its prediction error and gradient are used to weigh incorrectly classified observations, facilitating the reduction of error in the next tree at each step. Both extreme gradient boosting (Xgboost) and gradient boosting method (GBM) algorithms use gradient across trees to reduce prediction errors by learning from previous tree errors.

#### Feed-forward neural network

A neural network is constructed from layers of data matrices that interconnect with each other to classify a target variable like the neuronal networks of brains. Among diverse types of neural network algorithms, feed-forward neural network is a model whose direction of data processing only moves forward, i.e., the connection between the nodes does not form a cycle. It uses various regularization algorithms, such as L1 and L2 regularization and dropout ratio, to prevent overfitting.

naive Bayes: naive Bayes algorithms classify observation based on Bayes Theorem. The assumption of independence of features is mandatory. For binary outcomes, Bernoulli naive Bayes is applied. By knowing prior probability of target variable, *P(A)*, and predictor feature, P(B), are independent of each other. *P(B|A)* (also known as likelihood) is the current conditional probability of occurring predictor feature when the target outcome has occurred. We can calculate the probability of occurrence of target B when predictor feature A has occurred *P(A|B)* (i.e., posterior probability) according to Equation (3) (reference).


(3) $$\begin{array}{l} P\left(A|B\right)=\frac{P\left(B|A\right).P\left(A\right)}{P\left(B\right)}\; \end{array}$$

#### Stacked ensemble algorithms

One of the methods to improve the performance of different machine learning algorithms is combining results of more than one algorithm prediction. Then, by using combined predicted values to train one metalearner algorithm on top of all other algorithms, different kinds of algorithms can be selected for metalearner or base learner (even bagging and boosting ensemble algorithms like random forest and XGBoost).

In summary, these machine learning algorithms have been used in this study: RF, GBM, XGB, FNN, GLM, and stacked ensemble with GLM metalearner (EnsGLM), and stacked ensemble with naive Bayes metalearner (EnsNB).

### Dataset splitting

After assessing inclusion and exclusion criteria, 416 patients were included for machine learning analysis. This dataset was split into two parts, stratified by target variable (MACE); one part contained 80% of total data, used for hyperparameters optimization, training, and performance analysis, and 20% as a hold-out set to assess algorithms validation on an unseen data at the end of the analysis. In summary, of total 416 included patients, train group included 331 patients (MACE/Total = 36/331 or 10.8%) and test (hold-out) group included 84 patients (MACE/Total = 10/84 or 11.9%).

### Pre-processing

▸ Before splitting the data set, the following steps were taken for data pre-processing:

Variables with more than 30% missing values, such as “vulnerable plaque” and “plaque characterization,” were dropped from the dataset. Features related to drugs were dropped due to uncertainty according to patients' history and potential inconsistency of drug compliance, the dropped features include beta-blocker, ACE/ARB, statin, and ASA/Clopidogrel. There was uncertainty about the time of data gathering and the burden of dysrhythmia at the time of image acquisition, so variables related to heart rate and rhythm were dropped. (They included premature ventricular contraction, premature atrial complex, normal sinus rhythm, and atrial fibrillation.)

▸After splitting the data set, the following steps were taken for data pre-processing:

Among clinical variables, renal failure was excluded due to its near zero variance. Among anatomical variables, the following variables were excluded due to near zero variance: percentage of PDA, PLB, and distal LAD stenosis and plaque type of distal LAD, PDA, and PLB. No variable had zero variance. Variables with less than 30% missing values were EF (with 25.9% missing values) and BMI (with 1.95% missing values). To prevent data leakage between the train and the test sets, missing value imputations were conducted separately for the train and test set with the bagging algorithm method with 500 numbers of sequential trees (bagImpute method in H_2_O packages).

Collinearity between numeric variables was assessed in the train dataset by correlation matrix and correlation plot (Supplementary Table S1 and Supplementary Figure S2). There was no significant collinearity, considering Pearson's R correlation coefficient (|r|) > 0.7, between numeric variables (19).

To stabilize variance among numeric variables and make their distribution more Gaussian-like, data power transformation with the Yeo–Johnson method for positive values (Equation 4) was used (20).


(4) $$\psi \left(\lambda ,y\right)=\{\;\begin{array}{c} \frac{\left({\left(y+1\right)}^{\lambda }-1\right)}{\lambda }if\lambda \neq 0,y\geq 0\\ \log\left(y+1\right)if\lambda =0,y\geq 0 \end{array}\;$$

For positive values in this study, it is somehow equivalent to box–cox transformation. To find λ, maximum likelihood estimation is used during pre-processing. We extracted estimated lambda values to facilitate the reverse transformation of variables after training to improve interpretability (Supplementary Table S2).

To make normalized numeric variables, all their values were centered to their means (Equation 5, Centering values), and scaled to their standard deviation to have zero mean and standard deviation of one (Equation 6, Scaling centered values to standard deviation) (21).


(5) $$\begin{array}{l} \bar{x_{ij}}=x_{ij}-\bar{x_{i}}\; \end{array}$$

(6) $$\begin{array}{l} \bar{x_{ij}}=\frac{x_{ij}-\bar{x_{i}}}{sd_{i}}\; \end{array}$$

All categorical variables were converted to dummy variables by one-hot encoding, with each category as a separate variable. For example, hypertension (HTN) with 0 and 1 categories for non-presence and presence, respectively, was converted to two separate variables of “HTN_X0” and “HTN_X1,” while each of them has 0 and 1 categories. This conversion was conducted due to the sensitivity of some ML algorithms such as neural networks to variable types as 0 or 1.

Z-scores, Yeo–Johnson transformed values, and their original values are shown in Supplementary Tables S3–S6.

### Resampling strategy

To find the best hyperparameters, assess models' performance, and avoid overfitting during the training process, 5-fold cross-validation was conducted with specified fold assignment to make all models be trained and tested on exactly the same populations.

### Tuning strategy

For each algorithm, its specific hyperparameters were optimized by a random search method, with 100 iterations as the terminating limit.

A random forest with 1,000 parallel random decision trees was developed. The number of random variables in each split was set to three. Minimum numbers of observation in each terminal node were set to nine. The maximum depth of each tree was set to 18 to prevent overfitting and creating a sophisticated model that learns every aspect of data.

A GBM model consisting of 1,000 consecutive decision trees was created. The maximum depth of each tree was set to 8, and the minimum number of observations in each terminal node was set to 16. The sample rate for each consecutive tree was set to random 0.8 of the total number of observations. To avoid terminating algorithm before detecting global minima, the learning rate of 0.01 was selected to adjust the amount of weight modification between consecutive trees.

An XGBoost model consisting of 5,000 consecutive trees was created. The maximum depth of each tree was set to 13. The minimal number of observations in each terminal node was set to 11. To avoid the algorithm being stuck in local minima and to prevent premature model fitting, a learning rate of 0.2 was used in the weight adjustment of consecutive trees. A sampling rate of 0.7 was selected, which selects a random 0.7 portion of the total number of observations in each consecutive tree.

XGBoost has improved the computation speed compared to GBM due to its specific programming design and its capability of parallel processing. In addition, it provides many regularization hyperparameters to reduce overfitting. Two of these well-known hyperparameters are L1 (alpha) and L2 (lambda) and their values range from 0 to infinity. Another regularization hyperparameter provided by the XGBoost algorithm is gamma. It is considered a pseudo-regularization hyperparameter that specifies a minimum amount of loss reduction required for further splitting each tree node. It is implemented after growing a tree. This method prunes leaves that do not meet the loss reduction minimum. Another hyperparameter provided by both XGBoost and GBM algorithms is the dropout ratio, which is well-known for its implementation in neural networks. For tree-based algorithms, it is sometimes referred to as a dropout additive regression tree (DART) (22). To prevent the model from becoming too complex, DART randomly drops out a predefined ratio of trees in boosting sequence.

We used regularization terms alpha and lambda (similar to L1 and L2 for neural network) with values 0.1 and 0.1, respectively, to implement a penalty for wrongly predicted values and to avoid overfitting. Min child's weight was set to 11. The drop rate was set to 0.5.

A GLM model for binary outcome by logistic regression (LR) algorithm was created. The alpha hyperparameter was set to zero which indicated applying ridge penalty or L2 regularization. Using the ridge penalty, we attempted to reduce overfitting and, in particular, the variance of coefficients by penalizing larger values. Ridge penalty or shrinkage tries to penalize the estimated coefficients by adding a penalty term to the loss function. In the LR algorithm, maximizing log-likelihood is used to achieve the best value of each coefficient. The amount of penalty or shrinkage is controlled by a value of the λ parameter which is calculated during tunning. By employing this method, coefficients with larger values would be penalized more, and their values tend to decrease more than smaller-sized coefficients (Equation 7) (22, 23).


(7) $$\begin{array}{l} minimize\;\left(L\left(\beta \right)-\frac{\lambda }{2}\overset{K}{\underset{k=1}{\sum }}\beta_{k}^{2}\right)\; \end{array}$$

where β_*k*_, *k* = 1, ..., *K* are regression coefficients set and *L*(β) is the maximized log-likelihood of coefficients.

A feed-forward neural network was created with three hidden layers, each containing 16 fully connected neurons and 10 epochs. Input layer consisted of all input variables, with an input dropout ratio of 0.2 to reduce overfitting by selecting 80% of the total observations in each iteration. The learning rate of 0.001 was used to prevent a rapid decreasing of error rate to avoid the local minima trap; this, in turn, increases the likelihood of detecting global minima. The hyperbolic tangent function was selected as the activation function for each layer. To further avoid overfitting, L1 and L2 regularization with values of 0.01 and 0.1 were applied, respectively. A momentum start value of 0.2 was used to prevent the algorithm to be stuck in local minima.

Results of the best hyperparameters for each algorithm are shown in Supplementary Table S7.

### Performance assessment

To assess the performance of each algorithm, we use the AUC of the ROC. To compare between algorithm AUC values, we used the DeLong method.

### Variable importance

The results of the final training on the train set (80% of the total data) were used for variable importance analysis. We performed a permutation-based method to select the top important variables, determined by the change in the model's accuracy before and after permuting each variable. The mean variable importance score of each feature among all trees is calculated by Equation (8) (24).


(8) $$VI\left(X_{j}\right)=\frac{{\sum_{t=1}^{ntree}VI}^{\left(t\right)}\left(X_{j}\right)}{ntree}$$

where $VI^{\left(t\right)}\left(X_{j}\right)$ is the mean variable importance of the variable *X*_*j*_in tree *t*.

### Partial dependence plot

Partial dependence plots for the top important variables of each model were graphed to determine their relationship with the target variable.

By using the mean and standard deviation of each variable after the Yeo–Johnson transformation, the final z-scores of each observation were reversed to Yeo–Johnson transformed values. Then by applying the inverse Yeo–Johnson transformation, original values were obtained for each z-score. By knowing the original values of each z-score, we are able to improve the interpretation of partial dependence graphs.

We used the following inverse Yeo–Johnson function for reversing the transformation of Yeo–Johnson transformed values (Equation 9) (20).


(9) $$\psi^{-1}\left(\lambda ,x\right)=\{\;\begin{array}{c} {\left(x\lambda +1\right)}^{\frac{1}{\lambda }}-1if\lambda \neq 0\\ e^{\left(x+1\right)}if\lambda =0 \end{array}\;$$

## Results

### Baseline characteristic

The average age was 60 ± 10 years, and 53.8% of patients were men. Dyslipidemia was the most common frequent clinical risk factor (61.4%). In total, 57.0, 28.8, 23.0, 17.0, and 38.4% of the population had hypertension, DM, cigarette smoking, renal failure, and a positive familial history of CAD, correspondingly. The most common indication for CCTA was stable ischemic heart disease (59%) and 41.0% for other purposes, such as preoperative assessment, arrhythmia, or syncope. There was a statistical correlation between patients' CACS and other scoring systems; the other scoring systems increased as the patients' CACS sub-groups go up (*P* < 0.001). The baseline demographic and clinical characteristics of participants based on their calcium score system are shown in Table 1.

### Early cardiac evaluation

Of the total 500 included participants, 46 patients underwent early coronary angiography in less than 90 days after CCTA (Table 2). These patients were not enrolled in our final analysis. Compared to the other group of patients, even in patients who experience the MACE, the early coronary angiography group has a higher score in all of the conventional scoring systems reviewed in our article (*P* < 0.001).

**Table 2:** Clinical outcomes of the patients who underwent coronary computed tomography angiography.

**Outcome type** **(*N* = 92)**		**Frequency (%)**
Major adverse cardiovascular events (*N* = 46)	Hospitalization for unstable angina or heart failure	25 (54.3%)
	Late revascularization	9 (19.6%)
	Percutaneous coronary intervention	8 (17.4%)
	Coronary artery bypass graft	1 (2.2%)
	All-cause deaths	9 (19.6%)
	Non-fatal myocardial infarction	3 (6.5%)
Early event (*N* = 46)	Early coronary angiography	28 (60.9%)
	Early revascularization	18 (39.1%)
	Percutaneous coronary intervention	10 (21.7%)
	Coronary artery bypass graft	8 (17.4%)

### Outcomes and follow-up

Among the 454 patients, 38 patients were not enrolled due to missing the follow-up process (Figure 1). Of the remaining 416 participants, MACE was developed in 46 (11.0%) patients. The most common MACE was hospitalization which was seen in 25 (54.3%) patients. The median follow-up time was 20.5 ± 7.9 months. Outcomes details are shown in Table 2.

### ML risk prediction methods vs. conventional risk prediction methods

**Figure 2:**
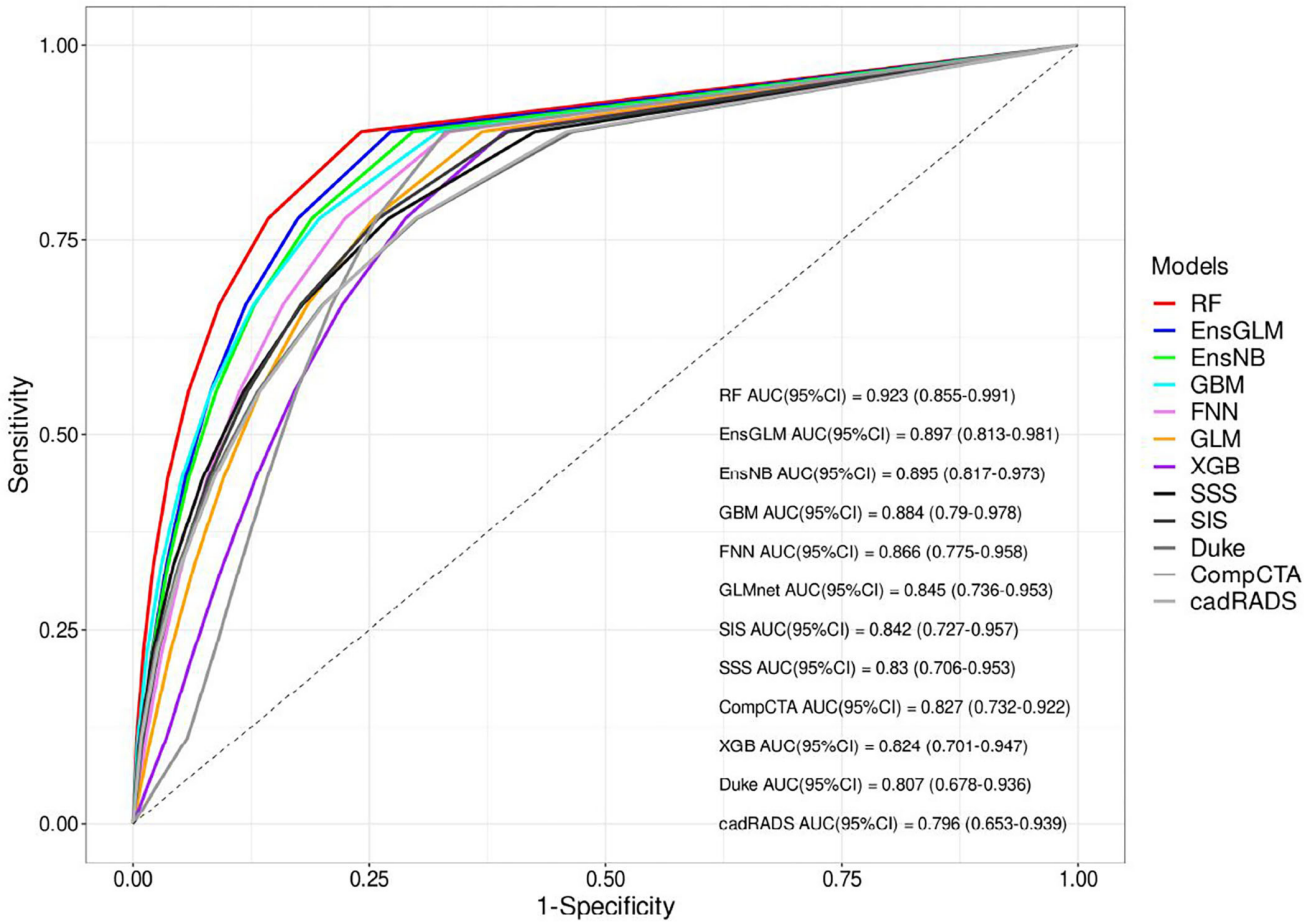
The receiver operating characteristic (ROC) curves of all models for unseen test set. AUC, area under the curve, CAD-RADS, coronary artery disease reporting and data system, Comp.CTAS, comprehensive computed tomography angiography score, EnsGLM, stacked ensemble with generalized linear model metalearner, EnsNB, stacked ensemble with naive Bayes metalearner, FNN, feed-forward neural network, GBM, Gradient Boosting Machine, GLM, generalized linear model, RF, random forest, SIS, segment involvement score, SSS, segment stenosis score, XGB, eXtreme Gradient Boosting.

Finally, 416 patients were enrolled to develop ML models and run the final analysis. Five machine learning algorithms have been done in this study. We used two ensemble methods that use all five machine learning models to increase the accuracy of the analysis. The results of cross-validation are shown in Table 3. Compared to the unseen test, the difference between AUCs values shows that our analysis has a good validation, so no overestimation has occurred. RF had the most AUC (0.92). Between the other conventional scores, the AUC for SIS was higher compared to the others (AUC:0.84). The AUCs are compared in Figure 2, Table 3, and Supplementary Table S8.

**Table 3:** Results of all models for predicting major adverse cardiovascular events in the patients who underwent coronary computed tomography angiography.

**Model**	AUC* **(95% confidence interval)**		**Threshold**	**Specificity**	**Sensitivity**
	**Cross-validation data**	**Unseen test set**			
RF**	0.79 (0.73–0.85)	0.92 (0.85–0.99)	0.16	0.77	1.0
EnsGLM***	0.78 (0.71–0.84)	0.90 (0.81–0.98)	0.21	0.85	0.8
EnsNB^$^	0.75 (0.69–0.80)	0.89 (0.82–0.97)	0.08	0.73	1.0
GBM^$$^	0.72 (0.64–0.80)	0.88 (0.79–0.98)	0.03	0.76	0.9
FNN^$$$^	0.79 (0.73–0.85)	0.87 (0.77–0.96)	0.09	0.65	1.0
GLM^#^	0.748 (0.70–0.80)	0.84 (0.74–0.95)	0.12	0.73	0.9
XGB^##^	0.779 (0.71–0.84)	0.82 (0.70–0.95)	0.22	0.84	0.8
SIS^###^	-	0.84 (0.73–0.96)	5.50	0.77	0.8
SSS^∧^	-	0.83 (0.71–0.95)	2.50	0.65	0.9
Comp. CTAS^∧∧^	-	0.83 (0.73–0.92)	8.77	0.66	1.0
Duke	-	0.81 (0.68–0.94)	2.50	0.68	0.8
CAD-RADS^∧∧∧^	-	0.80 (0.65–0.94)	3.50	0.68	0.8

*AUC, area under the curve; ^∧∧^CAD-RADS, coronary artery disease reporting and data system; ^∧∧∧^Comp.CTAS, comprehensive computed tomography angiography score; ***EnsGLM, stacked ensemble with generalized linear model metalearner; ^$^EnsNB, stacked ensemble with naive Bayes metalearner; ^$$$^FNN, feed-forward neural network; ^$$^GBM, Gradient Boosting Machine; ^#^GLM, generalized linear model; **RF, random forest; ^###^SIS, segment involvement score; ^∧^SSS, segment stenosis score; ^##^XGB, eXtreme Gradient Boosting.

**Figure 3:**
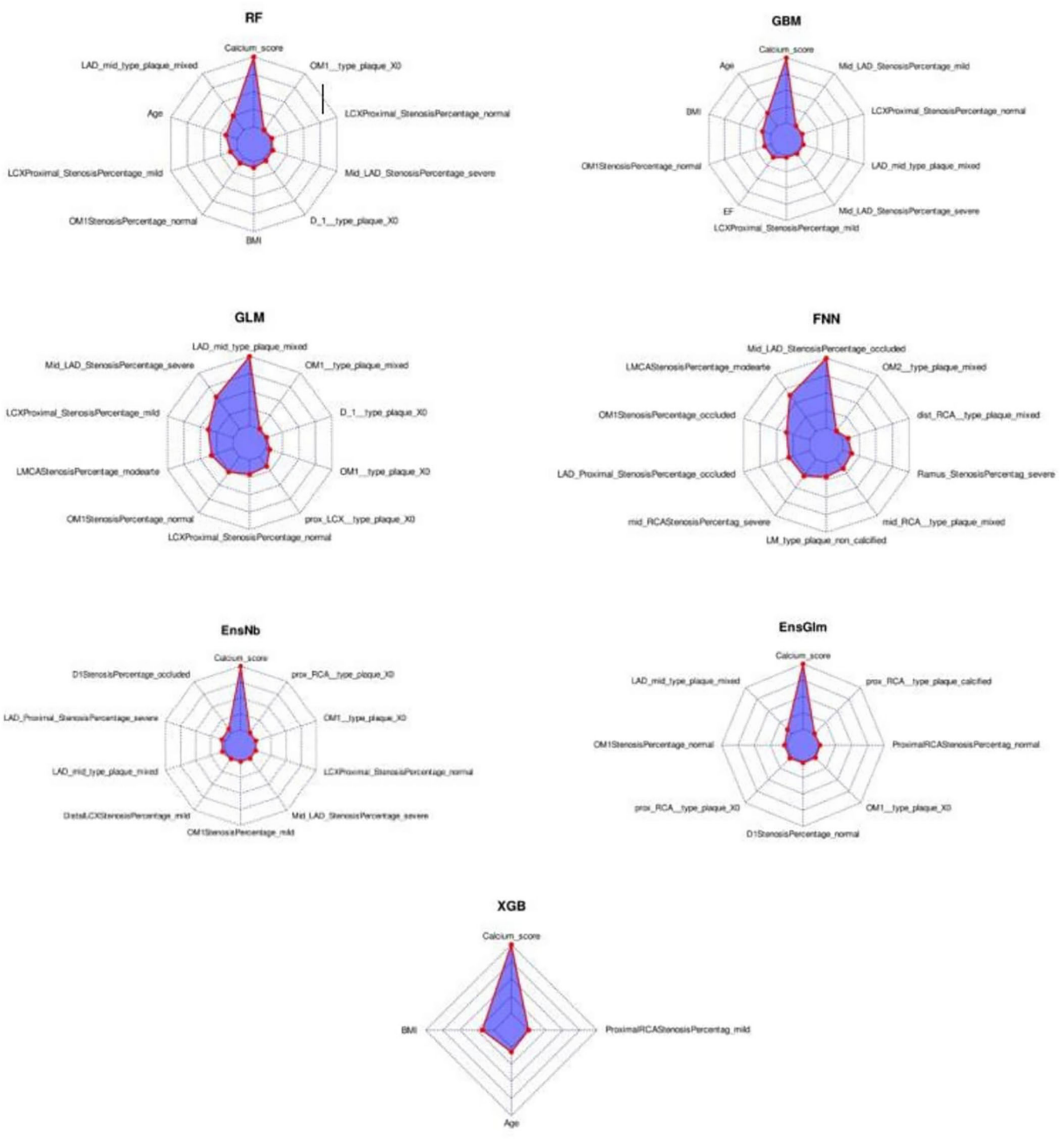
The variable importance radar-charts according to scaled importance of variables in the machine learning models. BMI, Body mass index, EnsGLM, stacked ensemble with generalized linear model metalearner, EnsNB, stacked ensemble with naive Bayes metalearner, FNN, feed-forward neural network, GBM, Gradient Boosting Machine, GLM, generalized linear model, LAD, left anterior descending artery, LCX, left circumflex artery, OM, obtuse marginal, RCA, right coronary artery, RF, random forest, XGB, eXtreme Gradient Boosting.

In the radar-chart, the scaled importance of variables in each model is shown, as it indicates that the calcium score plays a significant role in five of the ML algorithms (Figure 3). Anatomical variables have more impact on the models compared to the clinical index.

The partial dependence plots of the top variables of each ML model are shown in Supplementary Figures S2–S8. More anatomical features have been selected by ML algorithms as the top 10 variables compared to clinical features. The most important factor was CACS as an anatomical factor. The mean of CACS was about 130. Among clinical variables, BMI and age were selected by three algorithms, with the best rank of both in the second position. EF was selected by one algorithm (GBM). Original values of each standardized score in the partial dependence plot (PDP) were calculated by the reversal of pre-processing transformation. Among the top numeric variables of RF, the MACE rate increased from CACS 1 and reached its peak at about CACS 40–50. Then it decreased slightly and reached a plateau at about CACS 100. For other models in which CACS was included in top variables, the same trend is observable in PDP. For ages ranging from 56 to 69, MACE numbers increased rapidly; two plateaus also happened before and after this range. Between BMI of about 22 to 30, MACE numbers decreased. From a BMI of 30 to about 36, MACE increased and then it reached a plateau. The same pattern happened before the BMI of about 22. MACE trend had a relatively fixed downward slope from lowest to highest EF, except between 45 and 57% in which there is an increase then a decrease in MACE numbers. The variable frequency of selection and ranking is shown in Supplementary Table S9.

The list of all variables enrolled in the final analysis of all ML models is shown in Supplementary Table S10.

The comparison of clinical and coronary CCTA data between patients with and without MACE is shown in Table 4.

**Table 4:** The comparison of clinical and coronary computed tomography angiography data between patients with and without major adverse cardiovascular events (MACE).

**Variable**	**MACE (*N* = 46)**	**No MACE (*N* = 370)**	***P***
CACS*	256 (IQR: 100.5–413.5)	12 (IQR: 0–117)	*** <0.001***
Age (year)	64 (IQR: 59.25–70)	58 (IQR: 52–66)	***0.001***
BMI** (kg/m^2^)	28 (IQR: 25.01–30.11)	27.55 (IQR: 25.34–30.49)	0.804
Ejection fraction (%)	55 (IQR: 50–55)	55 (IQR: 55–60)	***0.043***
Gender			0.487
Female	24 (52.2%)	169 (45.7%)	
Male	22 (47.8%)	201 (54.3%)	
Diabetes mellitus [*N* (%)]	20 (43.5%)	94 (25.4%)	***0.016***
Hypertension [*N* (%)]	36 (78.3%)	201 (54.3%)	***0.004***
Dyslipidemia [*N* (%)]	34 (73.9%)	225 (60.8%)	0.122
Cigarette smoker [*N* (%)]	6 (13%)	64 (17.3%)	0.599
Family history of CAD^#^ [N (%)]	14 (30.4%)	146 (39.5%)	0.315
Renal failure [*N* (%)]	0 (0%)	18 (4.9%)	0.251
Comp. CTAS^##^	13 (IQR: 8.87–16.35)	6.75 (IQR: 3.3–11.3)	*** <0.001***
SSS^$^	5 (IQR: 3–7)	1 (IQR: 0–3)	*** <0.001***
SIS^$$^	6 (IQR: 4–8)	3 (IQR: 1–5)	*** <0.001***
Duke	4 (IQR: 3–5)	2 (IQR: 1–3)	*** <0.001***
CAD-RADS^∧^			*** <0.001***
0	0 (0%)	35 (9.5%)	
1	0 (0%)	14 (3.8%)	
2	9 (19.6%)	220 (59.5%)	
3	18 (39.1%)	64 (17.3%)	
4A	15 (32.6%)	29 (7.8%)	
4B	1 (2.2%)	3 (0.8%)	
5	3 (6.5%)	5 (1.3%)	

**BMI, body mass index, *CACS, calcium coronary artery score category, ^#^CAD, coronary artery disease, ^∧^CAD-RADS, coronary artery disease reporting and data system, ^##^Comp.CTAS, comprehensive computed tomography angiography score, ^$$^SIS, segment involvement score, and ^$^SSS, segment stenosis score. The bold and italic p-values indicate the statistically significant value (*p*-value < 0.05).

## Discussion

This study was designed to show the performance of the ML model for MACE prediction among patients undergoing CCTA for various reasons and also its capability to find important predictors of MACE among them. Part of the reason for this lack of research is the difficulties in dealing with artificial intelligence methods in survival and medical research, as well as their novelty. The first finding was that the ML scoring systems yielded better predictive performance than conventional scores. Among seven ML models consisting of clinical and anatomical data with CACS, RF had the highest efficacy in anticipating outcomes. Regarding the conventional scoring system, considering more factors than previous models, Comp.CTAS could not improve the performance of conventional scoring systems even though SIS has better performance (AUC: 0.84). Moreover, CACS as an anatomical characteristic was the robust MACE predictor in the majority of our ML models.

Based on our analysis, AUC for ML models showed better results, indicating the superiority of ML models (AUC: RF:0.92, EnsGLM:0.89), and it shows an improvement in MACE prediction performance over conventional CCTA scoring systems (AUC: 0.80–0.84). Among seven ML models, RF and EnsGLM have better performance in MACE prediction (AUC: RF: 0.92, EnsGLM: 0.89) than other ML models. XGB had the lowest performance among ML models (AUC: 0.82). These differences in models' performance could be due to their different inherent abilities to find a linear and non-linear relationship between predictors and target variables, their sensitivity to the data type, and between predictors' relationships. Although we tried to prevent factors from adversely influencing each algorithm by various pre-processing methods, most of the time this problem is inevitable to some degree. A similar study by Johnson et al. (10) used ML to develop a model to discriminate between patients with or without cardiovascular events. Compared to conventional scoring systems such as CAD-RADS, they realized that ML could better predict MACE occurrence (AUC: ML: 0.85, CAD-RADS: 0.75) in agreement with our study. Johnson's study employed only anatomical factors for developing the ML algorithm, whereas both anatomical and clinical factors along with CACS were considered in our ML models. In a recent study, Dan Li and his colleagues (25) used only clinical information to construct ML algorithms to predict CAD risk. They showed that ML-based risk stratification systems could be helpful in the prediction of CAD, although our study utilizes anatomical features, such as CACS, as well as clinical factors. In another study, several models were developed for MACE prediction regarding patients with suspected or established CAD who underwent CCTA. They indicated that adding CACS to the ML model achieves better performance (AUC = 0.88) compared to others (AUC: 0.68–0.77) (11). Our study went far beyond this study's findings based on demographic and clinical characteristics; we used CACS alongside both the above characteristics in our ML scoring systems. Moreover, compared to mentioned study, we employed more ML models comprising RF, GBM, FNN, GLM, EnsGLM, and EnsNB, as well as XGB, which was the only method used in the mentioned study. We found that other ML scoring systems were partially stronger than XGB. In Han's study, which was conducted in asymptomatic individuals during a checkup, 70 variables consisting of 35 clinical, 32 laboratories, and three CACS parameters were considered for analysis. The ML models' performance for the prediction of mortality was compared to three groups of conventional models (Framingham score+ CACS, atherosclerotic cardiovascular disease+ CACS, and logistic regression model). The AUC for ML was higher than the other models (0.82 vs. 0.70–0.79). The ML prediction models achieved better performance in mortality prediction (9). The strength of the mentioned study is that they considered both laboratory data and clinical data, which provides a better view of the clinical aspect of the participant. Our ML model consists of anatomical data with clinical factors, while in Han's study, anatomical data were not enrolled for analysis. Moreover, we designed both linear and non-linear ML models to better discover any correlation between the predictors and the target. In contrast, only the LogitBoosting was developed in Han's study.

The most consistent correlation among our ML models' variables with MACE was CACS in our study. This variable was listed by five algorithms among the top 10 important variables and ranked first in all of them. At the beginning part of the partial dependence plots for the CACS variable, more MACE have occurred with each increasing CACS unit till about 40–50, as we expected. Although, when the calcium score continues to increase, the MACE occurrence starts to decrease then becomes steady and forms a plateau in our diagram of about 100. Several studies have found that calcium score has a significant role in predicting CAD. For example, in Shoe's study, CACS > 400 can be a risk predictor of CAD (8). Sarwar et al. showed that CACS is the main predictor of cardiovascular events, and the absence of CAC is associated with a low risk of future cardiovascular events (2). Hypothetically, when CACS increases, coronary plaques are more stable, so the MACE occurrence does not continue to increase. Some studies, like Jinnouchi's study, indicated that lesions with dense calcifications are more likely to be stable plaques, while micro/punctate/fragmented calcifications are associated with either early-stage plaques or unstable lesions (26). Eventually, it shows that CACS positively correlates with MACE. However, in high CACS, this correlation reaches a plateau that may reflect the relatively benign nature of heavily calcified lesions due to their chronicity and replacement of active inflammation and lipid materials with calcified materials that lead to reduce the risk of acute events in these lesions.

Regarding variable importance, LAD mid-portion plaque was selected by five algorithms and ranked second in RF, first in GLM, second in EnsGLM, fourth in EnsNB, and eighth in GBM algorithms. In the partial dependence plot, more MACE was observed among mixed type plaque of LAD mid-portion compared to others. The severe LAD mid-portion stenosis variable was selected by four algorithms with second, seventh, eighth, and eighth positions in ranking. Those with severe LAD mid-portion had more MACE compared to the others.

Among clinical data, EF, age, and BMI were shown to be the most important variables in RF, GBM, and XGB methods. There was a rapidly increasing rate of MACE in the age range from 56 to 69. In line with our findings, the previous study showed that older patients (≥ 65 in male and ≥ 75 in female subjects) are at higher risk of MACE (27). MACE trend had a relatively fixed downward slope from lowest to highest EF, except between 45 and 57% in which, followed by an increase, there is a decrease in MACE. Our study confirmed the result of Son's study that more MACE was seen in patients with lower EF (28). In partial dependence plots of BMI, there was lower MACE in the BMI ranging from 22 to 30 kg/m^2^. Patients with BMI lower than 22 experienced more MACE compared to patients whose BMI is in the range of 22–30 kg/m^2^. Unlike previous factors, BMI plays a different role; it is called the obesity paradox, which indicates that there is a relationship between lower BMI and worse survival (29), the same findings are seen in our study.

Among the ML models applied in the current study, RF has a higher performance (AUC:0.92). Our RF model consists of anatomical variables (CACS, stenosis percentage and plaque type of LAD mid-portion, and stenosis percentage and plaque type of proximal LCX), and clinical variables (age and BMI). As shown in Figure 3, CACS plays a significant role in this model. RF is tree-based models consists of decision trees which are made up of nodes (30). New branches are created at each node of a decision tree by dividing the training samples along a particular feature and this process will decrease data heterogenicity (30). Moreover, RF is an ensemble technique made up of several decision tree classifiers, and RF is non-linear in nature. By voting or averaging the results across several classifiers, these techniques prevent overfitting (30). ML is being widely used in healthcare challenges, especially in the cardiovascular field (31–33). For example, the identification and forecasting of populations at high risk of unfavorable health outcomes, and the creation of proper interventions aimed at these populations are major uses of ML in public health (32). In several studies, the variables that had a significant impact on the CVD prediction were filtered out using the RF approach, and a prediction model was developed (33). RF has a lower chance of variance and overfitting of training data because the results of each of the decision trees that make up RF are averaged. In addition, RF is also scalable for big datasets and it can provide an estimation of what variables are significant in the classification. Based on RF advantages, it was shown to have the highest accuracy in disease and outcome prediction (34).

As mentioned above, the MACE prevalence in our study was 11.0%. According to previous studies such as Johnson et al. (10), MACE prevalence was 5.5%, even though our data were shown to be more balanced than theirs. In contrast to Johnson's study, the higher MACE prevalence in our study was due to including hospitalization as a MACE alongside other causes.

### Limitation

This study is limited by the fact that it was conducted with a small sample size. Further large-scale and long-term prospective multi-center studies including genetic and environmental factors as well as clinical and anatomical factors are needed to improve the ML-based scoring system for accurately predicting MACE.

We developed a web application according to our study population to predict MACE. The link for this web application can be found online at: https://behnam-hedayat.shinyapps.io/ctamace/.

## Conclusion

Consequently, the conventional scoring system could predict adverse events to some extent, and there is a need to incorporate artificial intelligence techniques to better predict MACE. Our study is the only one that adopted seven ML models consisting of both clinical and anatomical variables and revealed that the RF model has the best performance (AUC = 0.92) in MACE prediction compared to others. Anatomical predictors were selected more often than clinical predictors by ML algorithms as important variables contributing to models' accuracy. CACS was the major predictor of MACE in the models. Mixed-plaque of mid-portion of LAD was another predictor that consistently was among the top predictors. Among clinical variables, BMI, age, and EF had more impact on the accuracy of models in comparison to other clinical factors. It may be necessary to develop another ML method that includes more factors alongside clinical and anatomical features for MACE prediction.

## Data availability statement

The original contributions presented in the study are included in the article/Supplementary material, further inquiries can be directed to the corresponding author.

## Ethics statement

The studies involving human participants were reviewed and approved by Ethics Committee of Tehran Heart Center (Ethical Code: IR.TUMS.THC.REC.1399.022). The patients/participants provided their written informed consent to participate in this study.

## Author contributions

Conceptualization: NO and SG. Writing—original draft and visualization: SG, AF, and BH. Writing—review and editing: SG, AF, BH, NO, HC, and NB. Data gathering: SG, PA, and SS. Conventional statistical analysis: SG, AF, HM, and AJ. Machine learning analysis and web application development: BH. Supervision: NO, HC, and NB. All authors contributed to the article and approved the submitted version.

## Conflict of interest

The authors declare that the research was conducted in the absence of any commercial or financial relationships that could be construed as a potential conflict of interest.

## Publisher's note

All claims expressed in this article are solely those of the authors and do not necessarily represent those of their affiliated organizations, or those of the publisher, the editors and the reviewers. Any product that may be evaluated in this article, or claim that may be made by its manufacturer, is not guaranteed or endorsed by the publisher.

## Abbreviations

ACEI: Angiotensin-converting enzyme inhibitors
ARB: Angiotensin II receptor blockers
ASCVD: Atherosclerotic cardiovascular disease
AUC: Area under the curve
BMI: Body mass index
CABG: Coronary artery bypass graft surgery
CACS: Coronary artery calcium scoring
CAD: Coronary artery disease
CAD-RADS: Coronary artery disease reporting and data system
CCTA: Coronary computed tomography angiography
CI: Confidence intervals
Comp. CTAS: Comprehensive computed tomography angiography score
DM: Diabetes mellitus
EF: Ejection fraction
EnsGLM: Stacked ensemble with generalized linear model metalearner
EnsNB: Stacked ensemble with naive Bayes metalearner
FNN: Feed-forward neural network
GBM: Gradient boosting machine
GLM: Generalized linear model
ICA: Invasive coronary angiography
LAD: Left anterior descending artery
MACE: Major adverse cardiovascular events
MI: Myocardial infarction
ML: Machine learning
PCI: Percutaneous coronary intervention
RF: Random forest
SD: Standard deviation
SIS: Segment involvement score
SSS: Segment stenosis score
XGB: eXtreme Gradient Boosting.
