# Supplementary material for: Comparison of conventional scoring systems to machine learning models for the prediction of major adverse cardiovascular events in patients undergoing coronary computed tomography angiography
Source: Front Cardiovasc Med. 2022 Oct 26;9:994483. doi: 10.3389/fcvm.2022.994483 (PMC9643500; doi:10.3389/fcvm.2022.994483)
Supplement: Supplementary file 1 [file Data_Sheet_1.docx]

**“Supplementary Materials”**

**Comparison of Conventional Scores with Artificial Intelligence Models for Prediction of Major Adverse Cardiovascular Events in Patients Undergoing Coronary Computed Tomography Angiography**

**Short title:** Conventional vs. ML Scoring for Prediction of MACE Using CCTA

There is a common stenosis grading scale that is used in most stenotic scores (0=Normal: Absence of plaque and no luminal stenosis, 1=Minimal: Plaque with <25% stenosis, 2=Mild: 25%–49% stenosis, 3=Moderate: 50%–69% stenosis, 4=Severe: 70%–99% stenosis, 5=Occluded). (1)

Several conventional scores were used for the assessment of coronary arteries stenosis, which is described following: coronary artery calcium score (CACS), segment involvement score (SIS), segment stenosis score (SSS), Duke index, coronary artery disease reporting and data system (CAD-RADS), and comprehensive coronary computed tomography angiography score (Comp.CTAS). (2-6)

1) Coronary artery disease (CAD) vessel score: categorized into 5 groups, including normal, non-obstructive or mild (1-49% stenosis) CAD, single-vessel disease (SVD), two-vessel disease (2VD), and three-vessel disease (3VD)/ LM.

2) CAD severity: categorized into normal, non-obstructive CAD (1-49%), obstructive CAD (50% to 69%), sever obstructive CAD (more than 70%).

3) CACS, classified as five levels: zero, 1-10, 11-100, 101-400, and > 400.

4) SIS: was calculated as the total number of coronary artery segments exhibiting plaque, irrespective of the degree of luminal stenosis within each segment (minimum:0, maximum:16).

5) Segment stenosis score (SSS): Each individual coronary segment was graded as having no to severe plaque (0: no stenosis, 1: mild (1-49%) stenosis, 2: moderate (50-69%) stenosis, and severe (≥70%) stenosis) based on the extent of the obstruction of the coronary luminal diameter. Then, the extent scores of all 16 individual segments were summed to yield a total score ranging from 0 to 48.

6) Duke index: Group 0: No CAD; Group 1: ≥1 segment with 1–49% stenosis; Group 2: ≥2 segments with 1–49% stenosis and at ≥1 proximal segment with any stenosis; Group 3: ≥1 segment with 50–69% stenosis; Group 4: ≥2 segments with 50–69% stenosis or ≥1 segment with ≥70% stenosis; Group 5: ≥3 segments with 50–69% stenosis or ≥2 segments with ≥70% stenosis or proximal LAD with ≥70% stenosis; Group 6: ≥3 segments with ≥70% stenosis or ≥2 segments with ≥70% stenosis and proximal LAD with ≥70% stenosis); Group 7: LM with ≥50% stenosis.

7) CAD-RADS: CAD-RADS 0: absence of CAD; CAD-RADS 1: minimal non-obstructive CAD (1% to 24% maximal coronary stenosis or a plaque without stenosis); CAD-RADS 2: mild non-obstructive CAD (25% to 49% maximal coronary stenosis); CAD-RADS 3: moderate stenosis (50% to 69% maximal coronary stenosis); CAD-RADS 4A: severe stenosis 70% to 99% in 1 or 2 vessels; CAD-RADS 4b: sever stenosis 70% to 99% in 3 vessel or left main (LM) ≥50%; CAD-RADS 5: total coronary occlusion 100% coronary stenosis.

8) Comp.CTAS: ranging from 0 to 42 and divided into 3 groups: 0 to 5, 6 to 20, and >20, and was constructed based on the: 1) A 17-segment model of the coronary artery tree based on American Heart Association criteria; 2) literature describing the individual predictive value of plaque extent, severity, and composition variables as observed on coronary CTA; 3) The Leaman score which provides weight factors for plaque location.The programming language used for machine learning and survival analysis is R Version: 4.0.4 (2021-02-15).

# Packages of R used for machine learning and survival analysis including

**R packages:**

h2o(3.34.0.3), recipes(0.1.17), rsample (0.1.1), dplyr (1.0.7), rsample (0.1.1), forcats (0.5.1), pROC (1.18.0), data.table (1.14.2), VIM (6.1.1), knitr (1.37), lubridate (1.7.10), stringi (1.7.6), stringr (1.4.0), purr (0.3.4), tidyverse (1.3.1), survminer (0.4.9), survival (3.2.7), RCurl (1.98.1.5), fmsb (0.7.2)

# IDE used for analysis

**Integrated Development Environment:**

RStudio (1.4.1106): Integrated Development Environment for R. RStudio, PBC, Boston, MA URL <http://www.rstudio.com/>

We provide a link for the present study source code: https://github.com/hedayatbehnam/ctamace_study

In addition, we developed a web application (WebApp) according to our study population to predict MACE The link for this web application can be found online at: <https://behnam-hedayat.shinyapps.io/ctamace/> and the link for WebApp source code can be found online at: <https://github.com/hedayatbehnam/ctamace>

**Supplementary Table 1**. The Correlation Matrix of Included Numeric Variables in The Training Set

| Variable | CACS | Age | BMI | EF |
| --- | --- | --- | --- | --- |
| **CACS** | 1.0000000 | 0.2582534 | -0.0018758 | -0.1203230 |
| **Age** | 0.2582534 | 1.0000000 | -0.0594390 | -0.3057301 |
| **BMI** | -0.0018758 | -0.0594390 | 1.0000000 | 0.0745444 |
| **EF** | -0.1203230 | -0.3057301 | 0.0745444 | 1.0000000 |

BMI: body mass index, CACS: coronary artery calcium score, EF: ejection fraction


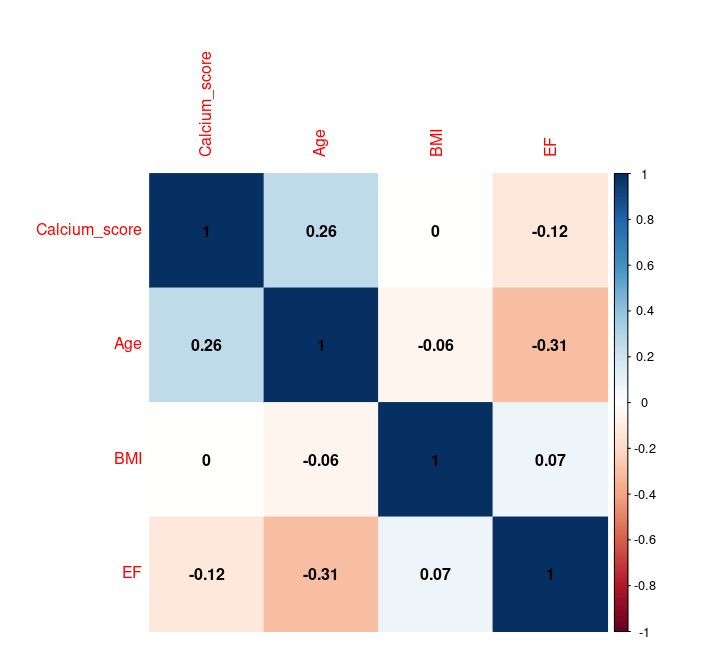


**Supplementary Figure 1.** Correlation Plot of Numeric Variables in The Training Set

^*^All Pearson R correlation coefficient (|r|) values were less than 0.3 which indicates weak collinearity between numeric variables.(7)

**Supplementary Table 2.** Estimated Maximum Likelihood of Lambda Parameter in Yeo-Johnson Transformation*

| CACS | Age | BMI | EF |
| --- | --- | --- | --- |
| 0.0009863345 | 0.4063089503 | 0.2593616622 | 4.4018371221 |

BMI: body mass index, CACS: coronary artery calcium score, EF: ejection fraction

* Limits of lambda were set to [-5,5].

**Supplementary Table 3.** Original Values of Coronary Artery Calcium Score For Each Z-score And Yeo-Johnson Value

| Z-score | Yeo-Johnson value | Original value |
| --- | --- | --- |
| -1 | 0.7 | 1.013266 |
| 0 | 3.1 | 21.093210 |
| 1 | 5.4 | 217.256309 |
| 2 | 7.8 | 2367.823535 |

**Supplementary Table 4.** Original Values of Age For Each Z-score And Yeo-Johnson Value

| Z-score | Yeo-Johnson value | Original value |
| --- | --- | --- |
| -2 | 8.8 | 41.21467 |
| -1 | 9.7 | 50.00899 |
| 0 | 10.5 | 58.66845 |
| 1 | 11.4 | 69.38856 |
| 2 | 12.3 | 81.17576 |
| 3 | 13.1 | 92.57451 |

**Supplementary Table 5.** Original Values of Body Mass Index For Each Z-score And Yeo-Johnson Value

| Z-score | Yeo-Johnson value | Original value |
| --- | --- | --- |
| -3 | 4.4 | 17.83137 |
| -2 | 4.7 | 20.60981 |
| -1 | 5.0 | 23.68083 |
| 0 | 5.4 | 28.26414 |
| 1 | 5.7 | 32.09399 |
| 2 | 6.0 | 36.28302 |
| 3 | 6.4 | 42.46387 |

**Supplementary Table 6.** Original Values of Ejection Fraction For Each Z-score And Yeo-Johnson Value

| Z-score | Yeo-Johnson value | Original value |
| --- | --- | --- |
| -2 | 4162496 | 43.66734 |
| -1 | 7912609 | 50.68506 |
| 0 | 11662722 | 55.44689 |
| 1 | 15412836 | 59.13776 |
| 2 | 19162949 | 62.18787 |
| 3 | 22913062 | 64.80629 |
| 4 | 4162496 | 67.11179 |

**Supplementary Table 7.** Optimized Hyperparameter values for each machine learning models

| **RF** | | **GBM** | | **XGB** | | **FNN** | | **GLM** | |
| --- | --- | --- | --- | --- | --- | --- | --- | --- | --- |
| ntree | 1000 | Ntree | 1000 | Ntree | 5000 | Number and size of hidden layers | (16,16,16) | alpha | 0 (ridge penalization) |
| mtry | 3 | min_rows | 16 | learning rate | 0.3 | input_dropout_ratio | 0.2 |  |  |
| nodesize | 9 | max_depth | 8 | min_rows | 11 | Learning rate | 0.001 |  |  |
| max_depth | 18 | sampling rate | 0.8 | max_depth | 13 | Activation Method | Hyperbolic Tangent (**Tanh**) |  |  |
|  |  |  |  | sample_rate | 0.7 | L1 regularization | 0.01 |  |  |
|  |  |  |  | ref_alpha | 0.1 | L2 regularization | 0.1 |  |  |
|  |  |  |  | min_split_improvement | 0.1 | Start momentum | 0.2 |  |  |
|  |  |  |  | reg_lambda | 0.1 | Stable momentum | 0 |  |  |
|  |  |  |  |  |  | Ramp momentum | 1e+07 |  |  |

FNN: Feed-Forward Neural Network, GBM: Gradient Boosting Machine, GLM: Generalized Linear Model, RF: Random Forest, XGB: eXtreme Gradient Boosting

**Supplementary Table 8.** Comparing all possible combinations of models, sorted by increasing P values

| **Combinations** | **ROC 1** | **ROC 2** | **P values** |
| --- | --- | --- | --- |
| **XGB vs. EnsGLM** | 0.82 (0.70 - 0.95) | 0.90 (0.81 - 0.98) | ***0.010*** |
| **RF vs. GLM** | 0.92 (0.85 - 0.99) | 0.84 (0.74 - 0.95) | ***0.024*** |
| **RF vs. XGB** | 0.92 (0.85 - 0.99) | 0.82 (0.70 - 0.95) | ***0.035*** |
| **Duke vs. RF** | 0.81 (0.68 - 0.94) | 0.92 (0.85 - 0.99) | **0.070** |
| **XGB vs. EnsNB** | 0.82 (0.70 - 0.95) | 0.89 (0.82 - 0.97) | **0.073** |
| **CAD-RADS vs. RF** | 0.80 (0.65 - 0.94) | 0.92 (0.85 - 0.99) | **0.076** |
| **Comp.CTA vs. RF** | 0.83 (0.73 - 0.92) | 0.92 (0.85 - 0.99) | **0.086** |
| **SSS vs. RF** | 0.83 (0.71 - 0.95) | 0.92 (0.85 - 0.99) | 0.113 |
| **GLM vs. EnsGLM** | 0.84 (0.74 - 0.95) | 0.90 (0.81 - 0.98) | 0.124 |
| **SIS vs. RF** | 0.84 (0.73 - 0.96) | 0.92 (0.85 - 0.99) | 0.128 |
| **RF vs. GBM** | 0.92 (0.85 - 0.99) | 0.88 (0.80 - 0.98) | 0.135 |
| **XGB vs. GBM** | 0.82 (0.70 - 0.95) | 0.88 (0.80 - 0.98) | 0.151 |
| **GLM vs. EnsNB** | 0.84 (0.74 - 0.95) | 0.89 (0.82 - 0.97) | 0.157 |
| **Duke vs. FNN** | 0.81 (0.68 - 0.94) | 0.87 (0.77 - 0.96) | 0.183 |
| **Duke vs. EnsNB** | 0.81 (0.68-0.94) | 0.89 (0.82 - 0.97) | 0.183 |
| **CAD-RADS vs. FNN** | 0.80 (0.65 - 0.94) | 0.87 (0.77 - 0.96) | 0.188 |
| **CAD-RADS vs. EnsNB** | 0.80 (0.65 - 0.94) | 0.89 (0.82 - 0.97) | 0.192 |
| **RF vs. FNN** | 0.92 (0.85 - 0.99) | 0.87 (0.77 - 0.96) | 0.219 |
| **RF vs. EnsNB** | 0.92 (0.85 - 0.99) | 0.89 (0.82 - 0.97) | 0.221 |
| **Comp.CTA vs. EnsGLM** | 0.83 (0.73 - 0.92) | 0.90 (0.81 - 0.98) | 0.225 |
| **CAD-RADS vs. EnsGLM** | 0.80 (0.65 - 0.94) | 0.90 (0.81 - 0.98) | 0.227 |
| **RF vs. EnsGLM** | 0.92 (0.85 - 0.99) | 0.90 (0.81 - 0.98) | 0.228 |
| **Duke vs. EnsGLM** | 0.801 (0.68 - 0.94) | 0.90 (0.81 - 0.98) | 0.232 |
| **SSS vs. EnsNB** | 0.83 (0.71 - 0.95) | 0.89 (0.82 - 0.97) | 0.237 |
| **Comp.CTA vs. EnsNB** | 0.83 (0.73 - 0.92) | 0.89 (0.82 - 0.97) | 0.243 |
| **CAD-RADS vs. GBM** | 0.80 (0.65 - 0.94) | 0.88 (0.79 - 0.98) | 0.246 |
| **Duke vs. GBM** | 0.81 (0.68 - 0.94) | 0.88 (0.79 - 0.98) | 0.264 |
| **SSS vs. EnsGLM** | 0.83 (0.71 - 0.95) | 0.90 (0.81 - 0.98) | 0.285 |
| **SIS vs. EnsNB** | 0.84 (0.73 - 0.96) | 0.89 (0.82 - 0.97) | 0.297 |
| **SIS vs. EnsGLM** | 0.84 (0.73 - 0.96) | 0.90 (0.81 - 0.98) | 0.302 |
| **GBM vs. GLM** | 0.88 (0.79 - 0.98) | 0.84 (0.74 - 0.95) | 0.331 |
| **Comp.CTA vs. GBM** | 0.83 (0.73 - 0.92) | 0.88 (0.79 - 0.98) | 0.405 |
| **SSS vs. GBM** | 0.83 (0.71 - 0.95) | 0.88 (0.79 - 0.98) | 0.438 |
| **SIS vs. GBM** | 0.84 (0.73 - 0.96) | 0.88 (0.79 - 0.98) | 0.502 |
| **FNN vs. EnsNB** | 0.87 (0.77 - 0.96) | 0.89 (0.82 - 0.97) | 0.524 |
| **SSS vs. FNN** | 0.83 (0.71 - 0.95) | 0.87 (0.77 - 0.96) | 0.551 |
| **Duke vs. CAD-RADS** | 0.81 (0.68 - 0.94) | 0.80 (0.65 - 0.94) | 0.567 |
| **CAD-RADS vs. GLM** | 0.80 (0.65 - 0.94) | 0.84 (0.74 - 0.95) | 0.568 |
| **FNN vs. EnsGLM** | 0.87 (0.77 - 0.96) | 0.90 (0.81 - 0.98) | 0.573 |
| **FNN vs. XGB** | 0.87 (0.77 - 0.96) | 0.82 (0.70 - 0.95) | 0.580 |
| **SIS vs. CAD-RADS** | 0.84 (0.73 - 0.96) | 0.80 (0.65 -0.94) | 0.590 |
| **Comp.CTA vs. FNN** | 0.83 (0.73 - 0.92) | 0.87 (0.77 - 0.96) | 0.598 |
| **GBM vs. EnsGLM** | 0.88 (0.80 - 0.98) | 0.90 (0.81 - 0.98) | 0.615 |
| **Duke vs. GLM** | 0.81 (0.68 - 0.94) | 0.84 (0.74 - 0.95) | 0.626 |
| **SSS vs. CAD-RADS** | 0.83 (0.71 - 0.95) | 0.80 (0.65 -0.94) | 0.636 |
| **SIS vs. Duke** | 0.84 (0.73 - 0.96) | 0.81 (0.68 - 0.94) | 0.647 |
| **XGB vs. GLM** | 0.82 (0.70 - 0.95) | 0.84 (0.74 - 0.95) | 0.657 |
| **SSS vs. SIS** | 0.83 (0.71 - 0.95) | 0.84 (0.73 - 0.96) | 0.692 |
| **SSS vs. Duke** | 0.83 (0.71 - 0.95) | 0.81 (0.68 - 0.94) | 0.709 |
| **SIS vs. FNN** | 0.84 (0.73 - 0.96) | 0.87 (0.77 -0.96) | 0.716 |
| **GBM vs. EnsNB** | 0.88 (0.79 - 0.98) | 0.89 (0.82 - 0.97) | 0.730 |
| **FNN vs. GLM** | 0.87 (0.77 - 0.96) | 0.84 (0.74 - 0.95) | 0.731 |
| **Comp.CTA vs. GLM** | 0.83 (0.73 - 0.92) | 0.84 (0.74 - 0.95) | 0.741 |
| **Comp.CTA vs. CAD-RADS** | 0.83 (0.73 - 0.92) | 0.80 (0.65 -0.94) | 0.751 |
| **SIS vs. Comp.CTA** | 0.84 (0.73 - 0.96) | 0.83 (0.73 - 0.92) | 0.753 |
| **FNN vs. GBM** | 0.87 (0.77 -0.96) | 0.88 (0.79 - 0.98) | 0.756 |
| **SSS vs. GLM** | 0.83 (0.71 - 0.95) | 0.84 (0.74 - 0.95) | 0.774 |
| **SIS vs. XGB** | 0.84 (0.73 - 0.96) | 0.82 (0.70 - 0.95) | 0.777 |
| **CAD-RADS vs. XGB** | 0.80 (0.65 - 0.94) | 0.82 (0.70 - 0.95) | 0.791 |
| **CAD-RADS vs. Duke** | 0.83 (0.73 - 0.92) | 0.81 (0.68 - 0.94) | 0.823 |
| **Duke vs. XGB** | 0.81 (0.68 - 0.94) | 0.82 (0.70 -0.95) | 0.861 |
| **EnsGLM vs. EnsNB** | 0.90 (0.81 - 0.98) | 0.89 (0.82 -0.97) | 0.886 |
| **SSS vs. XGB** | 0.83 (0.71 - 0.95) | 0.82 (0.70 -0.95) | 0.938 |
| **SIS vs. GLM** | 0.84 (0.73 - 0.96) | 0.84 (0.74 -0.95) | 0.939 |
| **Comp.CTA vs. XGB** | 0.83 (0.73 - 0.92) | 0.82 (0.70 -0.95) | 0.960 |
| **SSS vs. Comp.CTA** | 0.83 (0.71 - 0.95) | 0.83 (0.73 -0.92) | 0.966 |

RF: Random Forest, EnsGLM: Stacked ensemble with GLM metalearner, EnsNB: Stacked ensemble with naive-bayes metalearner, GBM: Gradient Boosting Machine, FNN: Feed-Forward Neural Network, GLM: Regularized Regression Models, XGB: Extended Gradient Boosting, SIS: segment involvement score, SSS: segment stenosis score, CAD-RADS: Coronary artery disease reporting and data system, Comp.CTAS: comprehensive computed tomography angiography score

**
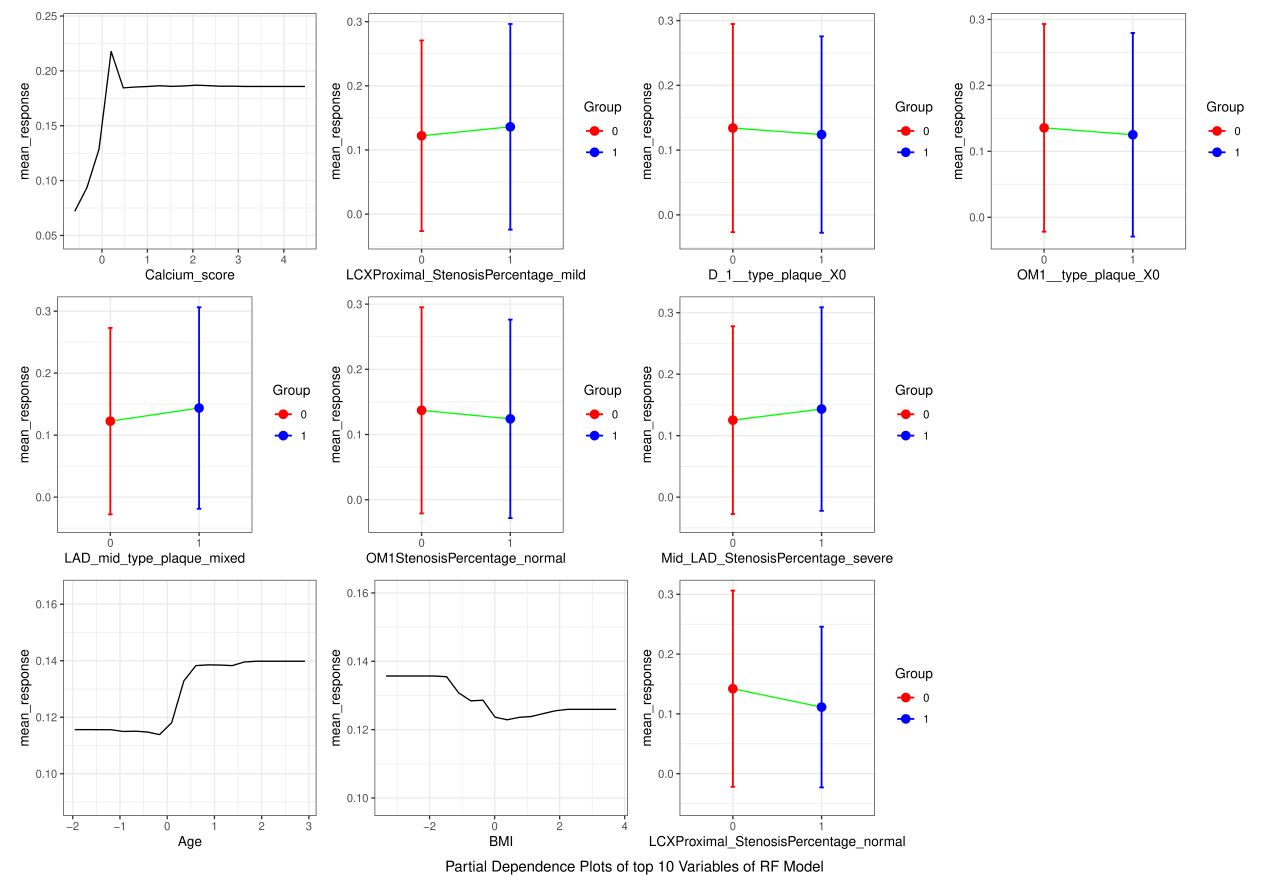
**

**Supplementary Figure 2.** Partial Dependence Plots of Top 10 Variables of The Random Forest (RF) Model

**
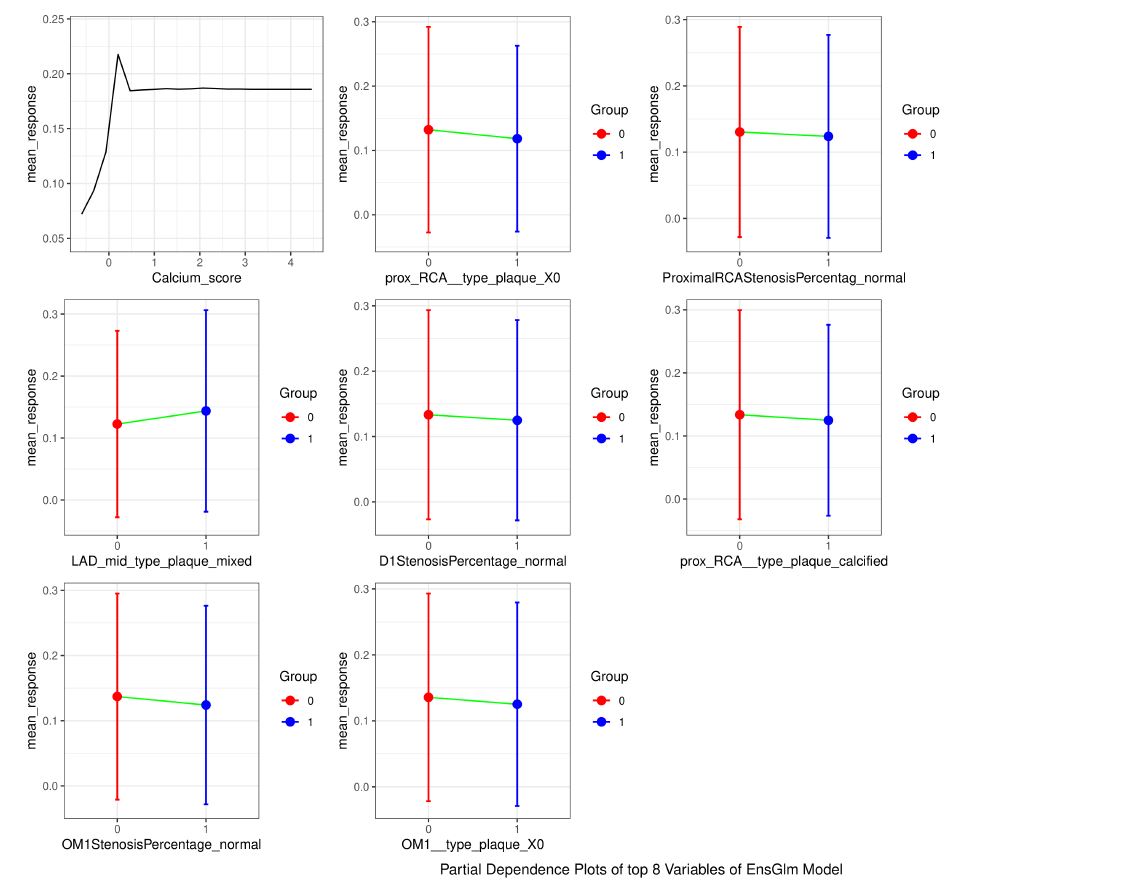
**

**Supplementary Figure 3.** Partial Dependence Plots of Top Eight Variables of The Stacked Ensemble with Generalized Linear Model Metalearner (EnsGLM) Model

**
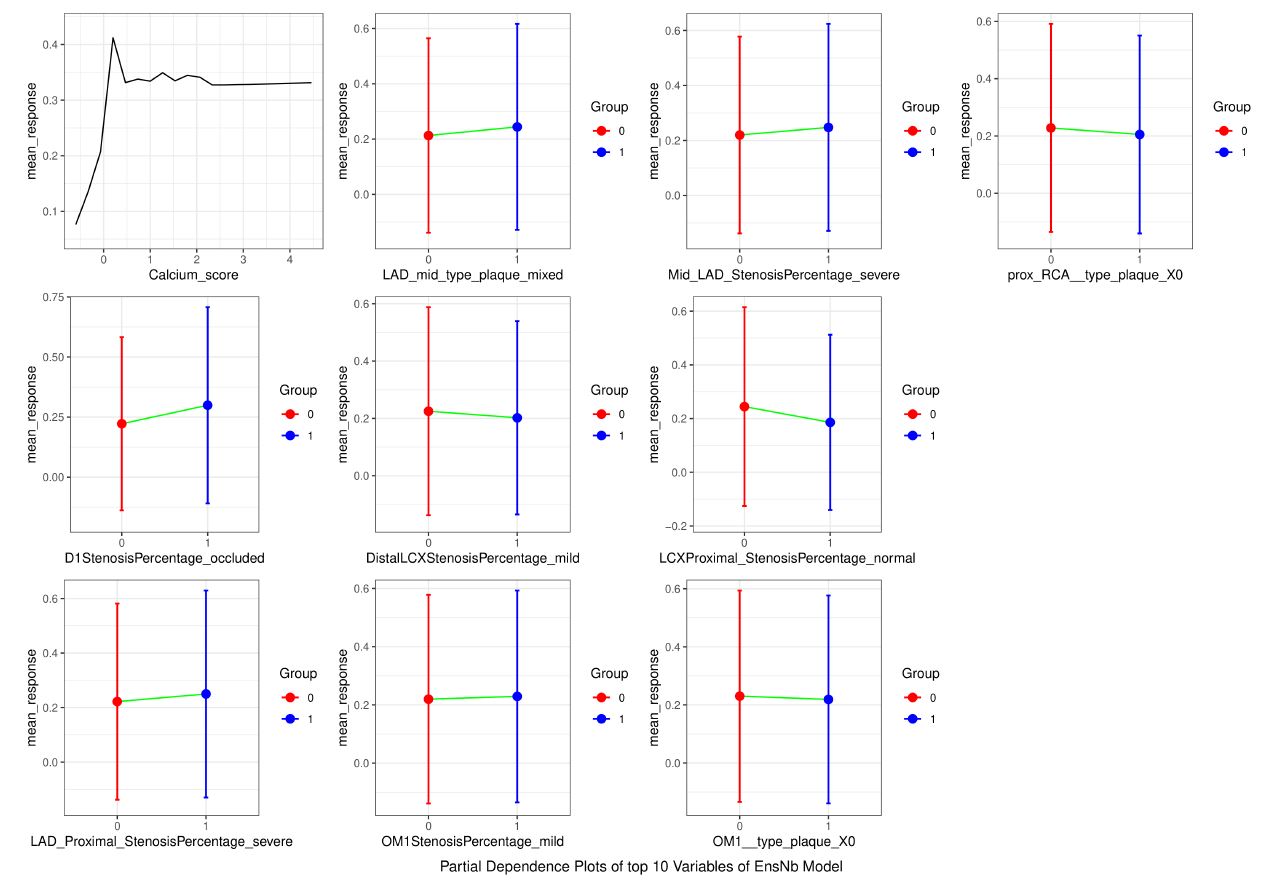
**

**Supplementary Figure 4.** Partial Dependence Plots of Top 10 Variables of The Stacked Ensemble with Naive-Bayes Metalearner (EnsNB) Model

**
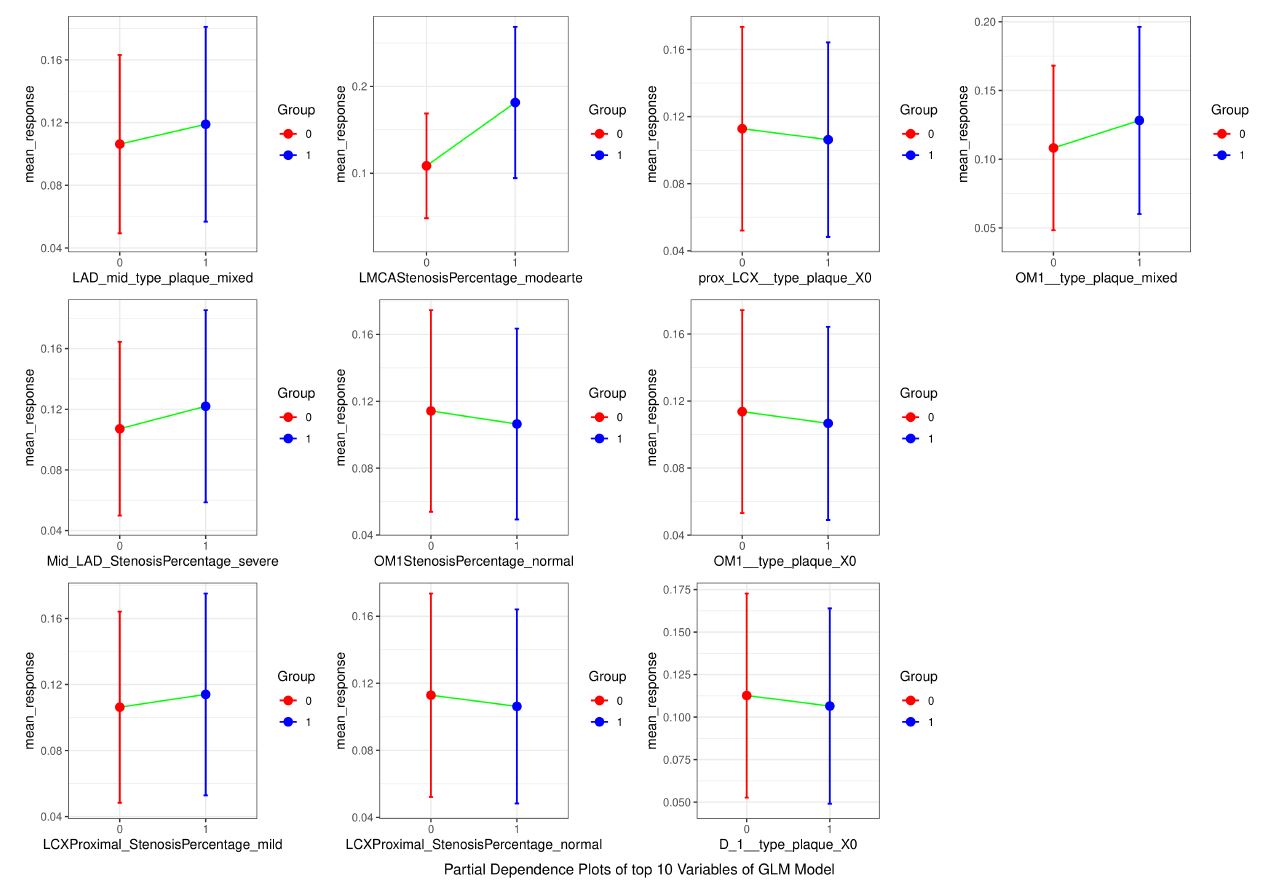
**

**Supplementary Figure 5.** Partial Dependence Plots of Top 10 Variables of The Generalized Linear Model (GLM) Model

**
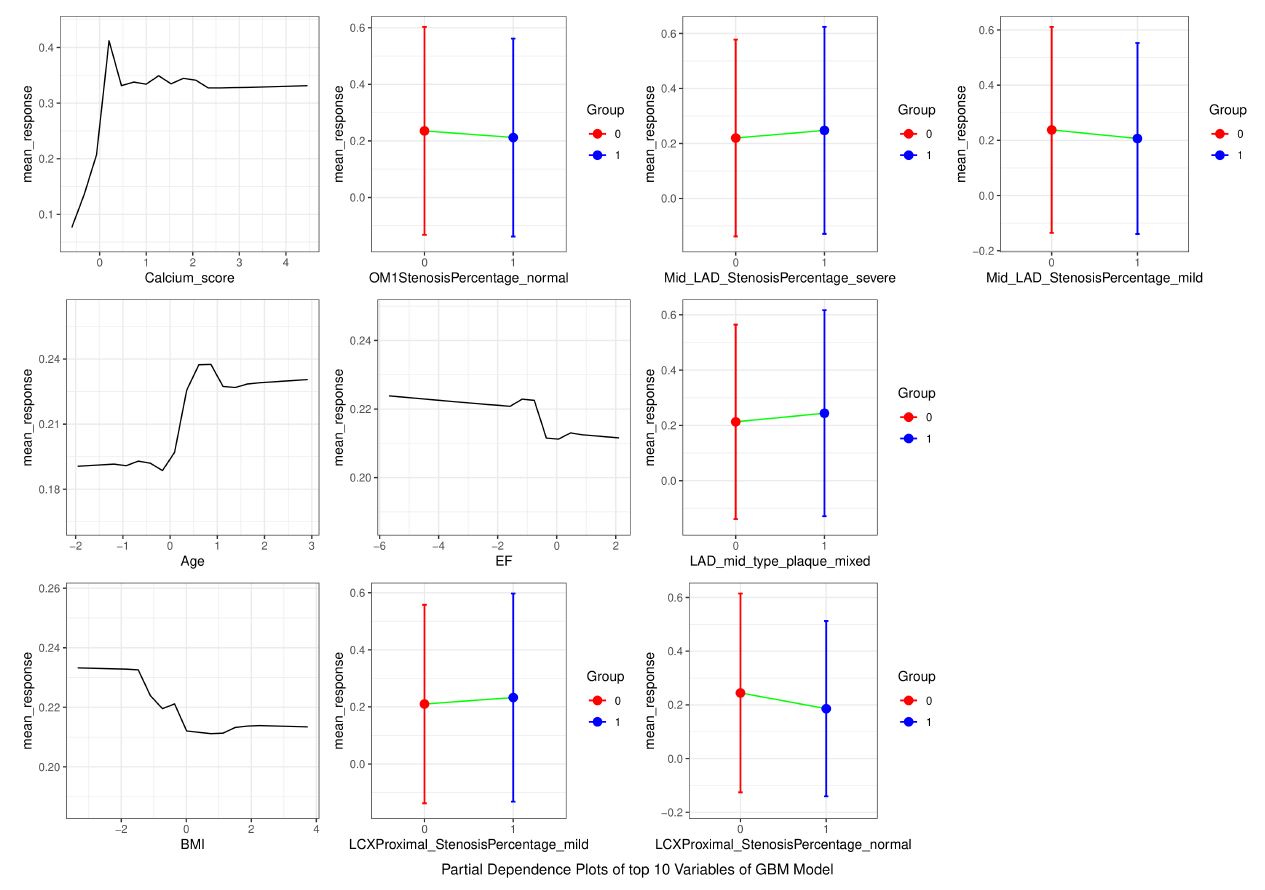
**

**Supplementary Figure 6.** Partial Dependence Plots of Top 10 Variables of The Gradient Boosting Machine (GBM) Model

**
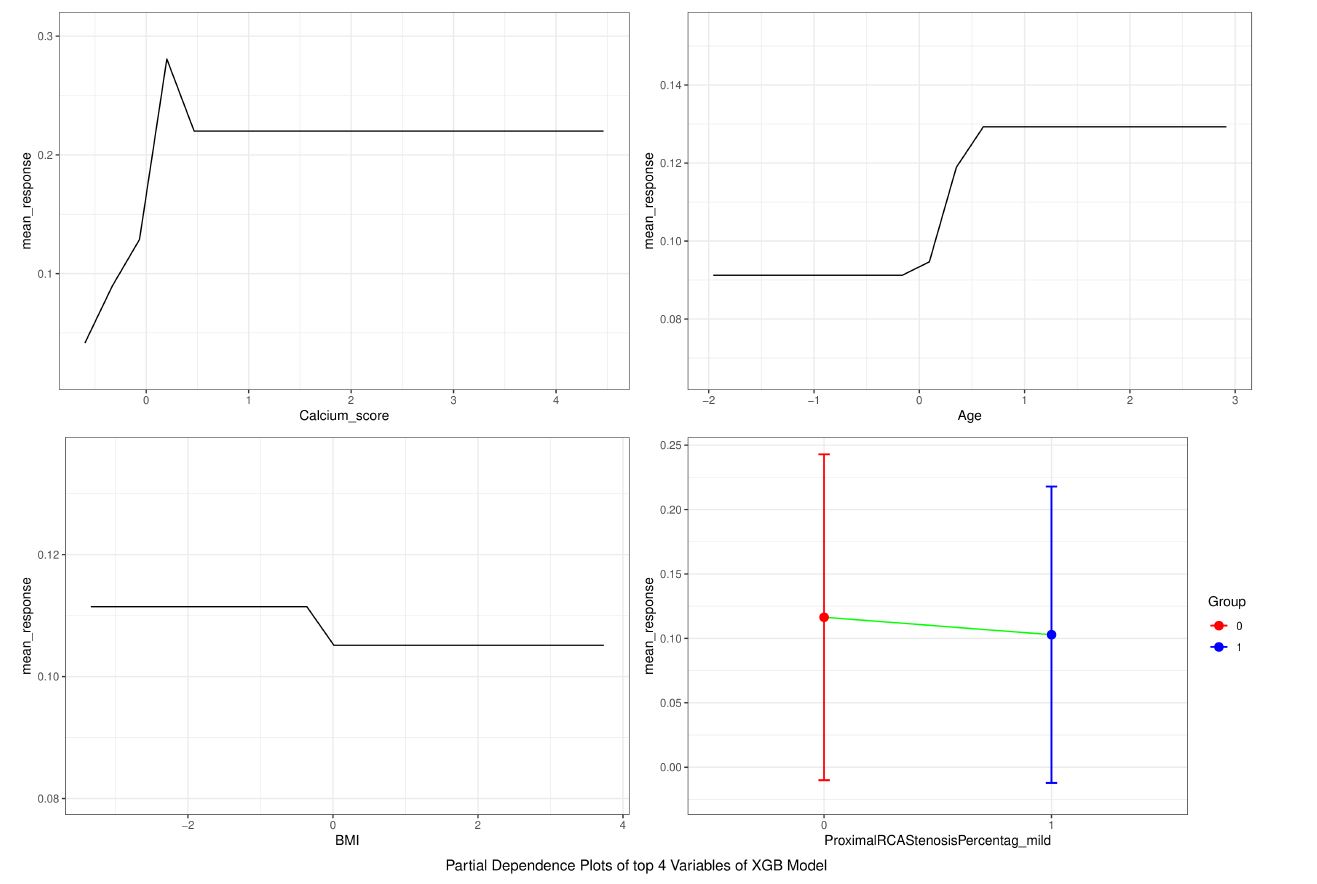
**

**Supplementary Figure 7.** Partial Dependence Plots of Top Four Variables of The eXtreme Gradient Boosting (XGB) Model

**
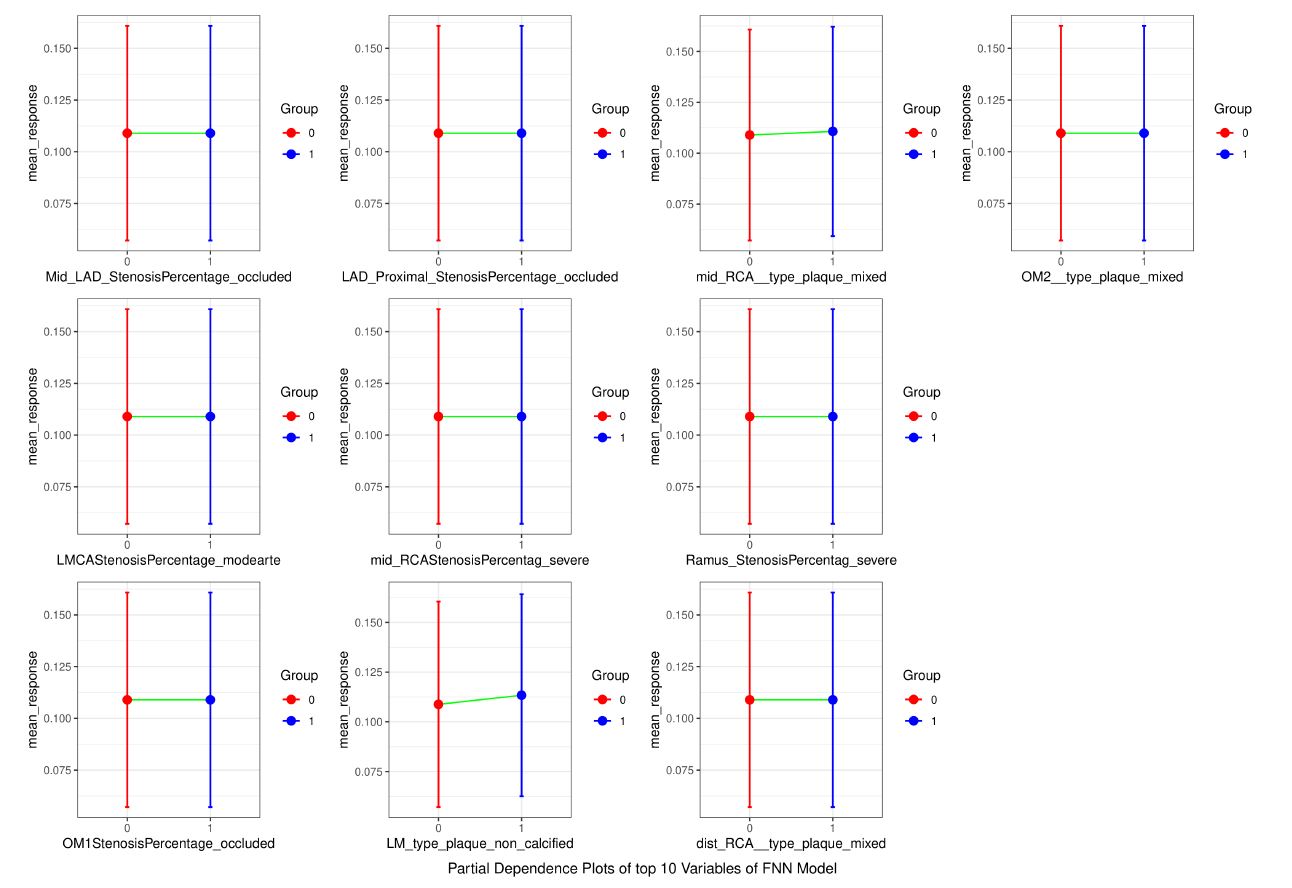
**

**Supplementary Figure 8.** Partial Dependence Plots of Top 10 Variables of The Feed-Forward Neural Network (FNN) Model

**Supplementary Table 9.** The Variables Frequency of Selection and Ranking

| **Variable** | **Algorithm** | **AUC** | **Frequency** | **Rank** |
| --- | --- | --- | --- | --- |
| Calcium_score | RF | 0.92 | 5 | 1 |
| Calcium_score | EnsGlm | 0.90 | 5 | 1 |
| Calcium_score | EnsNb | 0.89 | 5 | 1 |
| Calcium_score | GBM | 0.88 | 5 | 1 |
| LAD_mid_type_plaque_mixed | GLM | 0.84 | 5 | 1 |
| Calcium_score | XGB | 0.82 | 5 | 1 |
| LAD_mid_type_plaque_mixed | RF | 0.92 | 5 | 2 |
| LAD_mid_type_plaque_mixed | EnsGlm | 0.90 | 5 | 2 |
| LAD_mid_type_plaque_mixed | EnsNb | 0.89 | 5 | 4 |
| LAD_mid_type_plaque_mixed | GBM | 0.88 | 5 | 8 |
| Mid_LAD_StenosisPercentage_severe | GLM | 0.84 | 4 | 2 |
| BMI | XGB | 0.82 | 4 | 2 |
| OM1StenosisPercentage_normal | EnsGlm | 0.90 | 4 | 3 |
| BMI | GBM | 0.88 | 4 | 3 |
| OM1StenosisPercentage_normal | GBM | 0.88 | 4 | 4 |
| OM1StenosisPercentage_normal | RF | 0.92 | 4 | 5 |
| OM1StenosisPercentage_normal | GLM | 0.84 | 4 | 5 |
| BMI | RF | 0.92 | 4 | 6 |
| OM1__type_plaque_X0 | EnsGlm | 0.90 | 4 | 6 |
| LCXProximal_StenosisPercentage_normal | GLM | 0.84 | 4 | 6 |
| Mid_LAD_StenosisPercentage_severe | EnsNb | 0.89 | 4 | 7 |
| Mid_LAD_StenosisPercentage_severe | GBM | 0.88 | 4 | 7 |
| Mid_LAD_StenosisPercentage_severe | RF | 0.92 | 4 | 8 |
| LCXProximal_StenosisPercentage_normal | EnsNb | 0.89 | 4 | 8 |
| OM1__type_plaque_X0 | GLM | 0.84 | 4 | 8 |
| LCXProximal_StenosisPercentage_normal | RF | 0.92 | 4 | 9 |
| BMI | EnsGlm | 0.90 | 4 | 9 |
| OM1__type_plaque_X0 | EnsNb | 0.89 | 4 | 9 |
| LCXProximal_StenosisPercentage_normal | GBM | 0.88 | 4 | 9 |
| OM1__type_plaque_X0 | RF | 0.92 | 4 | 10 |
| Age | GBM | 0.88 | 3 | 2 |
| Age | RF | 0.92 | 3 | 3 |
| LCXProximal_StenosisPercentage_mild | GLM | 0.84 | 3 | 3 |
| Age | XGB | 0.82 | 3 | 3 |
| LCXProximal_StenosisPercentage_mild | RF | 0.92 | 3 | 4 |
| LCXProximal_StenosisPercentage_mild | GBM | 0.88 | 3 | 6 |
| LMCAStenosisPercentage_modearte | FNN | 0.87 | 2 | 2 |
| prox_RCA__type_plaque_X0 | EnsGlm | 0.90 | 2 | 4 |
| LMCAStenosisPercentage_modearte | GLM | 0.84 | 2 | 4 |
| D_1__type_plaque_X0 | RF | 0.92 | 2 | 7 |
| D_1__type_plaque_X0 | GLM | 0.84 | 2 | 9 |
| Prox_RCA__type_plaque_X0 | EnsNb | 0.89 | 2 | 10 |
| Mid_LAD_StenosisPercentage_occluded | FNN | 0.87 | 1 | 1 |
| D1StenosisPercentage_occluded | EnsNb | 0.89 | 1 | 2 |
| LAD_Proximal_StenosisPercentage_severe | EnsNb | 0.89 | 1 | 3 |
| OM1StenosisPercentage_occluded | FNN | 0.87 | 1 | 3 |
| LAD_Proximal_StenosisPercentage_occluded | FNN | 0.87 | 1 | 4 |
| ProximalRCAStenosisPercentag_mild | XGB | 0.82 | 1 | 4 |
| D1StenosisPercentage_normal | EnsGlm | 0.90 | 1 | 5 |
| DistalLCXStenosisPercentage_mild | EnsNb | 0.89 | 1 | 5 |
| EF | GBM | 0.88 | 1 | 5 |
| mid_RCAStenosisPercentag_severe | FNN | 0.87 | 1 | 5 |
| OM1StenosisPercentage_mild | EnsNb | 0.89 | 1 | 6 |
| LM_type_plaque_non_calcified | FNN | 0.87 | 1 | 6 |
| ProximalRCAStenosisPercentag_normal | EnsGlm | 0.90 | 1 | 7 |
| mid_RCA__type_plaque_mixed | FNN | 0.87 | 1 | 7 |
| prox_LCX__type_plaque_X0 | GLM | 0.84 | 1 | 7 |
| prox_RCA__type_plaque_calcified | EnsGlm | 0.90 | 1 | 8 |
| Ramus_StenosisPercentag_severe | FNN | 0.87 | 1 | 8 |
| dist_RCA__type_plaque_mixed | FNN | 0.87 | 1 | 9 |
| HTN_X1 | EnsGlm | 0.90 | 1 | 10 |
| Mid_LAD_StenosisPercentage_mild | GBM | 0.88 | 1 | 10 |
| OM2__type_plaque_mixed | FNN | 0.87 | 1 | 10 |
| OM1__type_plaque_mixed | GLM | 0.84 | 1 | 10 |

BMI: body mass index, HTN: Hypertension, RF: Random Forest, EnsGLM: Stacked Ensemble with Generalized Linear Model metalearner, EnsNB: Stacked Ensemble with Naive-Bayes metalearner, GBM: Gradient Boosting Machine, FNN: Feed-Forward Neural Network, GLM: Generalized Linear Model, XGB: eXtreme Gradient Boosting, LAD: left anterior descending artery, LCX: left circumflex, RCA: right coronary artery, OM: obtuse marginal, D: diagonal

**Supplementary Table 10.** The Variables Inserted to Final Analysis For Machine Learning Models

| **No.** | **Included Variable*** |
| --- | --- |
| 1 | Coronary artery calcium score |
| 2 | Age |
| 3 | Gender |
| 4 | Body mass index |
| 5 | Ejection fraction |
| 6 | Major adverse cardiac events |
| 7 | Diabetes mellitus |
| 8 | Hypertension |
| 9 | Dyslipidemia |
| 10 | Cigarette smoker |
| 11 | Family history of coronary artery disease |
| 12 | Stenosis Percentage of left main coronary artery |
| 13 | Stenosis Percentage of proximal part of left anterior descending artery |
| 14 | Stenosis Percentage of mid part of left anterior descending artery |
| 15 | Stenosis Percentage of diagonal 1 |
| 16 | Stenosis Percentage of diagonal 2 |
| 17 | Stenosis Percentage of proximal part of left circumflex artery |
| 18 | Stenosis Percentage of distal part of left circumflex artery |
| 19 | Stenosis Percentage of obtuse marginal artery 1 |
| 20 | Stenosis Percentage of obtuse marginal artery 2 |
| 21 | Stenosis Percentage of proximal part of right coronary artery |
| 22 | Stenosis Percentage of mid part of right coronary artery |
| 23 | Stenosis Percentage of distal part of right coronary artery |
| 24 | Plaque type of left main coronary artery |
| 25 | Plaque type of proximal part of left anterior descending artery |
| 26 | Plaque type of mid part of left anterior descending artery |
| 27 | Plaque type of diagonal 1 |
| 28 | Plaque type of diagonal 2 |
| 29 | Plaque type of proximal part of left circumflex artery |
| 30 | Plaque type of distal part of left circumflex artery |
| 31 | Plaque type of obtuse marginal artery 1 |
| 32 | Plaque type of obtuse marginal artery 2 |
| 33 | Plaque type of proximal part of right coronary artery |
| 34 | Plaque type of mid part of right coronary artery |
| 35 | Plaque type of distal part of right coronary artery |
| 36 | Dominancy of coronary arteries |

* Categorical variables presented as dummy variables

**References**

1. Leipsic J, Abbara S, Achenbach S, Cury R, Earls JP, Mancini GJ, et al. SCCT guidelines for the interpretation and reporting of coronary CT angiography: a report of the Society of Cardiovascular Computed Tomography Guidelines Committee. Journal of cardiovascular computed tomography. 2014;8(5):342-58.

2. Min JK, Shaw LJ, Devereux RB, Okin PM, Weinsaft JW, Russo DJ, et al. Prognostic value of multidetector coronary computed tomographic angiography for prediction of all-cause mortality. Journal of the American College of Cardiology. 2007;50(12):1161-70.

3. Xie JX, Cury RC, Leipsic J, Crim MT, Berman DS, Gransar H, et al. The coronary artery disease–reporting and data system (CAD-RADS) prognostic and clinical implications associated with standardized coronary computed tomography angiography reporting. JACC: Cardiovascular Imaging. 2018;11(1):78-89.

4. Min JK, Berman DS, Dunning A, Achenbach S, Al-Mallah M, Budoff MJ, et al. All-cause mortality benefit of coronary revascularization vs. medical therapy in patients without known coronary artery disease undergoing coronary computed tomographic angiography: results from CONFIRM (COronary CT Angiography EvaluatioN For Clinical Outcomes: An InteRnational Multicenter Registry). European heart journal. 2012;33(24):3088-97.

5. Rana JS, Dunning A, Achenbach S, Al-Mallah M, Budoff MJ, Cademartiri F, et al. Differences in prevalence, extent, severity, and prognosis of coronary artery disease among patients with and without diabetes undergoing coronary computed tomography angiography: results from 10,110 individuals from the CONFIRM (COronary CT Angiography EvaluatioN For Clinical Outcomes): an InteRnational Multicenter Registry. Diabetes care. 2012;35(8):1787-94.

6. van Rosendael AR, Shaw LJ, Xie JX, Dimitriu-Leen AC, Smit JM, Scholte AJ, et al. Superior risk stratification with coronary computed tomography angiography using a comprehensive atherosclerotic risk score. JACC: Cardiovascular Imaging. 2019;12(10):1987-97.

7. Vatcheva KP, Lee M, McCormick JB, Rahbar MH. Multicollinearity in Regression Analyses Conducted in Epidemiologic Studies. Epidemiology (Sunnyvale, Calif). 2016;6(2).
